# PRMT7-Mediated PTEN Activation Enhances Bone Regeneration in Female Mice

**DOI:** 10.3390/ijms26072981

**Published:** 2025-03-25

**Authors:** Yingfei Zhang, Jia Qing, Yang Li, Xin Gao, Dazhuang Lu, Yiyang Wang, Lanxin Gu, Hui Zhang, Zechuan Li, Xu Wang, Yongsheng Zhou, Ping Zhang

**Affiliations:** 1 Department of Prosthodontics, Peking University Hospital of Stomatology, Beijing 100081, China; zyfdentist@163.com (Y.Z.); qingjia0723@163.com (J.Q.); 15896260251@163.com (Y.L.); gaox0724@163.com (X.G.); ludazhuang666@gmail.com (D.L.); wyiyang0116@163.com (Y.W.); green306@163.com (L.G.); zhanghui071023@163.com (H.Z.); 2311110622@stu.pku.edu.cn (Z.L.); 2011110543@bjmu.edu.cn (X.W.); 2 National Clinical Research Center for Oral Diseases, Peking University Hospital of Stomatology, Beijing 100081, China

**Keywords:** bone regeneration, gender dimorphism, epigenetics, H3R2me1, PTEN

## Abstract

Epigenetic regulation provides new insights into the mechanisms of osteogenic differentiation and identifies potential targets for treating bone-related diseases. However, the specific regulatory networks and mechanisms involved still need further investigation. In this study, we identify PRMT7 as a novel epigenetic regulator of mesenchymal stem cells (MSCs) osteogenic commitment. Conditional knockout of *Prmt7* in mice reveals a significant impairment in osteogenesis and bone regeneration, specifically in females, affecting both femurs and mandibles, with no noticeable effect in males. Mechanistically, PRMT7 modulates MSCs osteogenic differentiation by activating PTEN. Specifically, PRMT7 enhances PTEN transcription by increasing H3R2me1 levels at the PTEN promoter. Additionally, PRMT7 interacts with the PTEN protein and stabilizes nuclear PTEN, revealing an unprecedented pathway. Notably, overexpression of PTEN alleviates the osteogenic deficits observed in *Prmt7*-deficient mice. This research establishes PRMT7 as a potential therapeutic target for promoting bone formation/regeneration and offers novel molecular insights into the PRMT7–PTEN regulatory axis, underscoring its significance in bone biology and regenerative medicine.

## 1. Introduction

Epigenetic modifications, including DNA methylation and histone modifications, are essential in regulating cell pluripotency by modulating chromatin structure and influencing the transcription of genes critical for bone development [1]. Notably, histone lysine acetylation and methylation have been extensively studied for their roles in osteogenic differentiation [2,3]. While these modifications are well-established, the role of histone arginine methylation in osteogenesis remains a relatively unexplored field of research.

Histone and non-histone arginine methylation, mediated by protein arginine methyltransferases (PRMTs), are pivotal for various biological processes, including DNA repair, gene transcription, signal transduction, and stem cell fate determination [4]. Several studies, including our own, have highlighted the critical involvement of PRMTs in bone development. For example, PRMT1 is essential for RANKL-induced osteoclastogenesis and bone resorption [5]. PRMT3 promotes osteogenic differentiation by enhancing miR-3648 expression via increased H4R3me2a levels [6]. CARM1/PRMT4 facilitates osteogenic differentiation by modulating various H3 methylation sites [7]. Inhibition of PRMT5 has been shown to promote osteoblast differentiation while suppressing osteoclastogenesis [8,9]. Additionally, PRMT6 plays a positive role in osteogenesis, although the specific histone modifications involved have not been fully characterized [10]. These findings collectively underscore the importance of PRMTs in bone homeostasis.

Among the key players in bone regulation is PTEN, a well-known tumor suppressor that also governs numerous essential biological functions such as cell cycle control, migration, adhesion, DNA repair, stem cell maintenance, and metabolic regulation [11]. In bone marrow, *Pten* is indispensable for LepR^+^ cells to promote osteogenesis, while its inhibition suppresses osteogenic differentiation in dental pulp and bone marrow MSCs [12,13,14]. The expression of PTEN is tightly controlled at both the post-transcriptional and post-translational levels. Recent studies suggest that changes in histone acetylation and methylation at the PTEN promoter influence its transcription [15]. Moreover, post-translational modifications, such as phosphorylation [16], acetylation [17], methylation [18], and ubiquitination, are crucial for are crucial for regulating PTEN stability and activity, particularly in the context of cancer [19]. These modifications, especially in the context of cancer, collectively regulate PTEN’s function by controlling its stability, activity, and localization, playing a vital role in cellular processes such as proliferation, apoptosis, metabolism, and tumorigenesis.

In this study, we demonstrate that PRMT7 plays a key role in promoting bone formation and regeneration in the mandibles and femurs of female mice. We reveal that PRMT7 regulates osteogenesis by activating PTEN: it binds to the PTEN promoter and increases H3R2me1 levels, which in turn activates PTEN transcription. Additionally, PRMT7 stabilizes nuclear PTEN through direct interaction, enhancing its functional role in osteogenesis. Importantly, osteopenia caused by PRMT7 deletion in female mice can be rescued by PTEN overexpression, emphasizing the importance of PTEN in the PRMT7-mediated regulation of bone regeneration. In summary, our study uncovers a novel mechanism by which PRMT7 modulates osteogenesis through PTEN activation in female mice, providing a promising therapeutic target for bone regenerative medicine.

## 2. Results

### 2.1. Prmt7 CKO Mice Exhibit Impaired Bone and Dental Structures

**Figure 1:**
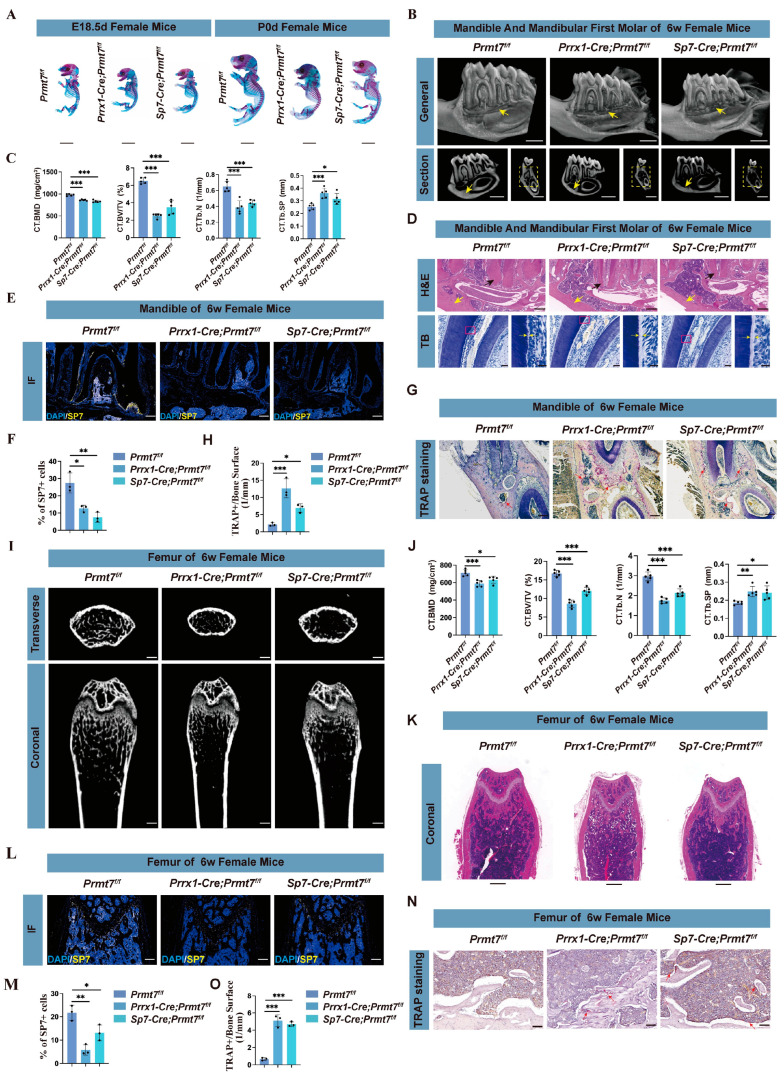
PRMT7 deficiency impairs bone and dental structures in female mice. (**A**) Alcian blue and alizarin red staining of E18.5 and P0 female control (*Prmt7^f/f^*) and CKO mice (*Prrx1-cre; Prmt7^f/f^* and *Sp7-cre; Prmt7^f/f^*). Scale bar: 5 mm. (**B**) Micro-CT analysis of the mandible in 6-week-old female control and CKO mice. The **upper** panel: 3D reconstructed sectional view of the mandible; the **lower left** panel: mesiodistal sectional view of the first molar; and the **lower right** panel: coronal sectional view of the first molar. The yellow arrow at the **upper** panel points to the bone around the apical area of the distal root of the first molar; the yellow arrow at the *lower left* panel points to the bone wall at the *lower border* of the mandibular body; and the yellow dashed box at the *lower right* panel indicates the bone between the furcation of the first molar and the mandibular canal. Scale bar: 1 mm. (**C**) Bone parameters quantitative analysis of the mandible in 6-week-old female control and CKO mice, including BMD, BV/TV, Tb. N, and Tb. Sp, obtained in B. (**D**) H&E staining (The **upper** panel) of the periapical bone in the mandible of 6-week-old female control and CKO mice. The black arrow indicates the cementum in the apical area of the distal root of the first molar, while the yellow arrow points to the bone wall at the *lower border* of the mandible. Toluidine blue staining (the **lower** panel) of the dental tissues at the alveolar crest of the distal root of the first molar. The yellow arrow in the **lower right** points to the predentin, which is an enlarged view of the area within the pink box in the lower left. Scale bars: 200 μm (**upper**), 50 μm (**lower left**), and 20 μm (**lower right**). (**E**) Immunofluorescence staining of SP7 in the mandible of 6-week-old female control and CKO mice. Scale bar: 200 μm. (**F**) Quantitative analyses of the ratio of SP7^+^ cells to total cells in the mandible of 6-week-old female control and CKO mice. (**G**) TRAP staining was performed on the first molar region of the mandible in 6-week-old female CKO mice and their control group. The red arrow indicates osteoclasts within the bone tissue. Scale bar: 200 μm. (**H**) Quantitative analysis of the number of TRAP+ osteoclasts/bone surface (1/mm) in the mandible of 6-week-old female CKO mice and their control group. (**I**) Micro-CT analysis of femurs from 6-week-old female control littermates and CKO mice. The **upper** panel shows the cross-sectional view of the metaphysis, and the **lower** panel shows the coronal view of the metaphysis. Scale bar: 500 μm. (**J**) Quantitative analysis of bone parameters at the femoral metaphysis growth plate, including BMD, BV/TV, Tb. N, and Tb. Sp, obtained in I. (**K**) H&E staining of the coronal section of the femur in 6-week-old female control littermates and CKO mice. Scale bar: 500 μm. (**L**) Immunofluorescence staining of SP7 in the femur of 6-week-old female control and CKO mice. Scale bar: 200 μm. (**M**) Quantitative analyses of the ratio of SP7^+^ cells to total cells in the femur of 6-week-old female control and CKO mice. (**N**) TRAP staining was performed on the femur of 6-week-old female CKO mice and their control group. The red arrow indicates osteoclasts within the bone tissue. Scale bar: 100 μm. (**O**) Quantitative analysis of the number of TRAP+ osteoclasts/bone surface (1/mm) in the femur of 6-week-old female CKO mice and their control group. Data were expressed as mean ± standard deviation (SD) and analyzed by one-way ANOVA. ns—no significant; * *p* < 0.05; ** *p* < 0.01; *** *p* < 0.001. For all CT analyses, the sample size in each group was n = 5. The sample size for immunofluorescence and TRAP quantification was n = 3 mice per genotype.

To investigate the role of PRMT7 in bone formation in vivo, we established Prmt7 conditional knockout (CKO) mouse models using Prrx1-Cre and Sp7-Cre mice (Figure S1A,B). At 6 weeks, female CKO mice (Prrx1-cre; Prmt7^f/f^ and Sp7-cre; Prmt7^f/f^) exhibited growth restriction compared to control littermates (Prmt7^f/f^), a phenotype not observed in males (Figure S1C). Alizarin red and alcian blue staining revealed significantly delayed bone formation in female CKO mice at E18.5 and P0, with no such differences in males (Figure 1A; Figure S1D). Micro-CT of the mandibles showed that in CKO mice, compared to the control group, there is a reduction in bone mass in the region between the apical area of the first molar root of the mandible and the mandibular nerve canal, with thinning of the mandibular bone wall, alongside decreased bone mineral density (BMD), bone volume (BV/TV), trabecular number (Tb. N), and increased trabecular space (Tb. Sp) (Figure 1B,C). H&E staining indicated CKO mice showed reduced bone mass, thinner cementum in the apical region, and a thinner lateral bone wall in the mandible compared to the control group, while toluidine blue staining showed widened predentin, indicating impaired dentin mineralization in female CKO mice (Figure 1D). Immunofluorescence results showed that PRMT7 knockdown in mice led to a decrease in SP7^+^ cells compared to the control group, suggesting that PRMT7 affects osteoblast maturation and activity (Figure 1E,F). Tartrate-resistant acid phosphatase (TRAP) staining showed a significant increase in TRAP-positive osteoclasts in the mandible of CKO mice compared to controls. This suggests that bone loss caused by PRMT7 deficiency may be associated with osteoclastogenesis (Figure 1G,H). However, no significant changes were observed in male mice (Figure S1E–K). We conducted the same experiment in the femur, and the results showed that PRMT7 deficiency also led to bone loss in the femurs of female mice. However, this phenomenon was not observed in male mice (Figure 1I–O; Figure S1L–R).

To assess bone regeneration, we created mandible and tibial injury models. Control mice showed nearly complete healing, while CKO female mice retained large defects, confirmed by lower bone mass in the defect area (Figure S2A–C,G–I). Male mice displayed no significant changes in regeneration capacity (Figure S2D–F,J–L). In summary, PRMT7 deficiency impairs bone and dental structures and hinders bone regeneration, with effects observed exclusively in females.

### 2.2. PRMT7 Regulates Osteogenic Differentiation in a Methyltransferase Activity-Dependent Manner

**Figure 2:**
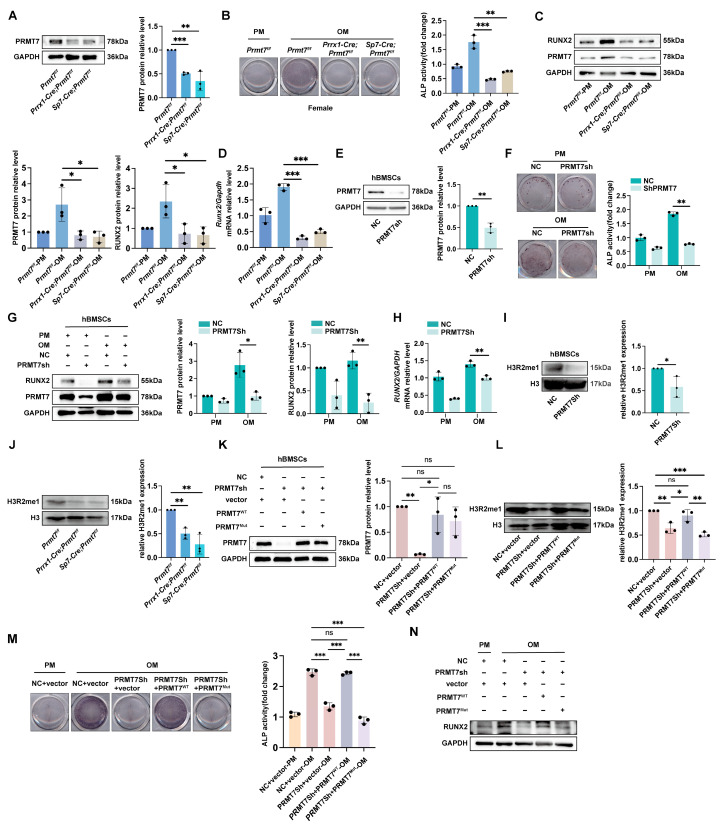
PRMT7 regulates osteogenic differentiation via its methyltransferase activity. (**A**) Representative Western blot images (**left**) of PRMT7 in 6-week-old female control littermates and CKO mice BMSCs. Quantification (**right**) of relative PRMT7 levels normalized to GAPDH. (**B**) Staining and quantification of ALP in 6-week-old female control littermates and CKO mice BMSCs after 7 days of osteogenic induction. PM—proliferation medium; OM—osteogenic induction medium. (**C**) Representative Western blot images of PRMT7 and RUNX2 in 6-week-old female control littermates and CKO mice BMSCs after 7 days of osteogenic induction. Quantification (the leftmost part of the next row) of relative PRMT7 and RUNX2 levels normalized to GAPDH. (**D**) qRT-PCR results of *Runx2* in 6-week-old female control littermates and CKO mice BMSCs after 7 days of osteogenic induction. (**E**) Representative Western blot images (**left**) of PRMT7 in hBMSCs after transfection with PRMT7sh and control lentivirus, respectively. Quantification (**right**) of relative PRMT7 levels normalized to GAPDH. (**F**) Staining and quantification of ALP in PRMT7sh and control hBMSCs after 7 days of osteogenic induction. (**G**) Representative Western blot images (**left**) of RUNX2 and PRMT7 in PRMT7sh and control hBMSCs after 7 days of osteogenic induction. Quantification (**right**) of relative RUNX2 and PRMT7 levels normalized to GAPDH. (**H**) qRT-PCR results of *RUNX2* in PRMT7sh and control hBMSCs after 7 days of osteogenic induction. (**I**) Representative Western blot images (**left**) of H3R2me1 in PRMT7sh and control hBMSCs after extracting nuclear proteins. Quantification (**right**) of relative H3R2me1 levels normalized to H3. (**J**) Representative Western blot images (**left**) of H3R2me1 in 6-week-old female control littermates and CKO mice BMSCs after extracting nuclear proteins. Quantification (**right**) of relative H3R2me1 levels normalized to H3. (**K**) Representative Western blot images (**left**) of PRMT7 in hBMSCs after transfection with lentivirus carrying either an empty vector, wildtype PRMT7 (PRMT7^WT^), or enzyme-mutant PRMT7 (PRMT7^Mut^). Quantification (**right**) of relative PRMT7 levels normalized to GAPDH. (**L**) Representative Western blot images (**left**) of H3R2me1 after transfection with lentivirus carrying either an empty vector, wildtype PRMT7 (PRMT7^WT^), or enzyme-mutant PRMT7 (PRMT7^Mut^) in hBMSCs and extraction of nuclear proteins. Quantification (**right**) of relative H3R2me1 levels normalized to H3. (**M**) Staining and quantification of ALP in hBMSCs after transfection with lentivirus carrying either an empty vector, wildtype PRMT7 (PRMT7^WT^), or enzyme-mutant PRMT7 (PRMT7^Mut^) and 7 days of osteogenic induction. (**N**) Representative Western blot images (**left**) of RUNX2 in hBMSCs after transfection with lentivirus carrying either an empty vector, wildtype PRMT7 (PRMT7^WT^), or enzyme-mutant PRMT7 (PRMT7^Mut^) and 7 days of osteogenic induction. n = 2 biological replicates. Unless otherwise specified, all experiments (including Western blot, qRT-PCR, and ALP assays) were performed with three independent biological replicates. All data are mean ± SD. (ns—not significant, *, *p* < 0.05, **, *p* < 0.01, ***, *p* < 0.001) (Independent samples *t*-test or one-way ANOVA).

To investigate the effect of PRMT7 on osteogenic differentiation of BMSCs, we extracted BMSCs from CKO and control mice for functional experiments. Western blot and qRT-PCR confirmed successful PRMT7 knockout (Figure 2A; Figure S3A). ALP activity analysis indicated a significant reduction in osteogenic differentiation of Prmt7-deficient female mBMSCs compared to controls (Figure 2B). However, the male mBMSCs showed no changes (Figure S3B). Additionally, RUNX2 expression, crucial for osteogenic differentiation, was significantly lower in *Prmt7*-deficient mBMSCs after induction (Figure 2C,D; Figure S3C). In human BMSCs (hBMSCs) undergoing osteogenic induction, PRMT7 protein and mRNA levels increased (Figure S3D,E). We established PRMT7 stable knockdown cells, confirming knockdown efficiency (Figure 2E; Figure S3F). After 7 days of osteogenic induction, PRMT7 knockdown cells showed reduced osteogenic differentiation, with lower RUNX2 levels compared to controls (Figure 2F–H; Figure S3G). Overall, PRMT7 enhances osteogenic differentiation of BMSCs in vitro.

To explore if PRMT7’s role in osteogenic differentiation is enzyme activity-dependent, we used a plasmid with mutations in the PRMT7 active site (E144A, D147A, E153A) [20]. PRMT7 is known to modulate H3R2me1 levels in MH-S cells [21]. Thus, we initially investigated whether the trends in H3R2me1 levels in BMSCs align with those observed in MH-S cells. Western blot analysis revealed that silencing PRMT7 resulted in a significant decrease in the global levels of H3R2me1 in MSCs (Figure 2I,J). Then, transfecting hBMSCs with lentivirus carrying wildtype (PRMT7^WT^) or mutant PRMT7 (PRMT7^Mut^) confirmed that H3R2me1 levels could not be restored by the mutant (Figure 2K,L). Osteogenic induction showed that PRMT7^WT^ restored differentiation ability in knockdown cells, while PRMT7^Mut^ did not (Figure 2M,N). Thus, PRMT7 promotes osteogenic differentiation of BMSCs in an enzyme activity-dependent manner.

### 2.3. PRMT7 Activates PTEN in Female Mice

**Figure 3:**
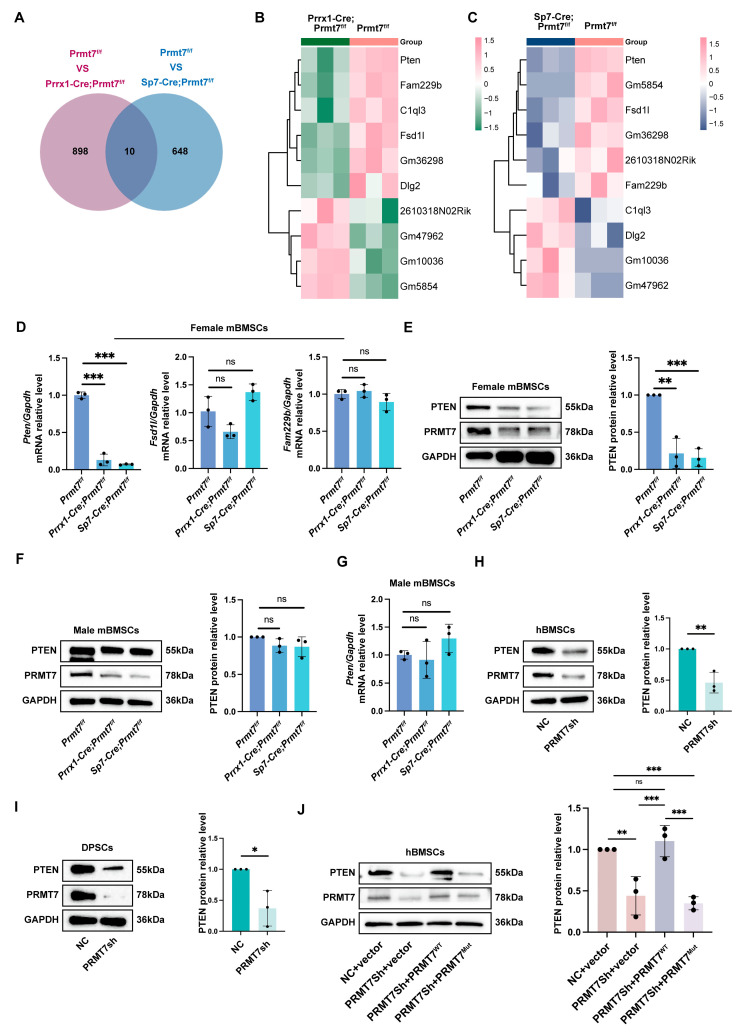
PRMT7 induces PTEN activation in female mice. (**A**) Venn diagram illustrating the overlap of differentially expressed genes between control and two strains CKO groups respectively, highlighting shared and unique genes in each group. (**B**) Heatmap displaying the expression levels of DEGs between *Prmt7^f/f^* and *Prrx1-Cre*; *Prmt7^f/f^* groups, with color intensity representing the level of gene expression across samples. (**C**) Heatmap displaying the expression levels of DEGs between *Prmt7^f/f^* and *Sp7-Cre*; *Prmt7^f/f^* groups, with color intensity representing the level of gene expression across samples. (**D**) qRT-PCR results of *Pten*, *Fsd1l*, and *Fam229b* in 6-week-old female control littermates and CKO mice BMSCs. (**E**) Representative Western blot images (**left**) of PTEN in 6-week-old female control littermates and CKO mice BMSCs. Quantification (**right**) of relative PTEN levels normalized to GAPDH. (**F**) Representative Western blot images (**left**) of PTEN in 6-week-old male control littermates and CKO mice BMSCs. Quantification (**right**) of relative PTEN levels normalized to GAPDH. (**G**) qRT-PCR results of *Pten* in 6-week-old male control littermates and CKO mice BMSCs. (**H**) Representative Western blot images (**left**) of PTEN in PRMT7sh and control hBMSCs. Quantification (**right**) of relative PTEN levels normalized to GAPDH. (**I**) Representative Western blot images (**left**) of PTEN in PRMT7sh and control DPSCs. Quantification (**right**) of relative PTEN levels normalized to GAPDH. (**J**) Representative Western blot images (**left**) of PTEN in hBMSCs after transfection with lentivirus carrying either an empty vector, wildtype PRMT7 (PRMT7^WT^), or enzyme-mutant PRMT7 (PRMT7^Mut^) and 7 days of osteogenic induction. Quantification (**right**) of relative PTEN levels normalized to GAPDH. All data are mean ± SD, n = 3 biological replicates. (ns—not significant, *, *p* < 0.05, **, *p* < 0.01, ***, *p* < 0.001) (Independent samples *t*-test or one-way ANOVA).

To investigate how PRMT7 regulates osteogenic differentiation, we performed RNA sequencing on mBMSCs from CKO mice compared to controls, identifying 354 down-regulated and 554 up-regulated genes in Prrx1-Cre; Prmt7^f/f^ mice. GO analysis revealed enrichment in regeneration and tooth mineralization, while KEGG highlighted the PI3K-AKT pathway (Figure S4A–C). In Sp7-Cre; Prmt7^f/f^ mice, we identified 658 differentially expressed genes (DEGs), mainly in the Wnt signaling pathway and mesenchymal cell differentiation (Figure S4D–F). Merging these DEGs yielded 10 genes visualized in heat maps (Figure 3A–C). Validation revealed only Pten consistently down-regulated (Figure 3D). In female mBMSCs, PTEN levels were significantly reduced, while male mBMSCs showed no change (Figure 3E–G), suggesting PTEN’s role in gender differences (Figure S4G,H). In hBMSCs and dental pulp stem cells (DPSCs), PRMT7 knockdown reduced PTEN levels (Figure 3H,I; Figure S4I,J), with overexpression showing the opposite (Figure S3K–N). Our results indicate that PRMT7 activates PTEN in an enzyme-dependent manner, confirmed using PRMT7^WT^ and PRMT7^Mut^ plasmids (Figure 3J). In summary, PRMT7 activates PTEN in female mice through enzymatic activity.

### 2.4. PRMT7 Regulates PTEN Stability and Transcriptional Activation

**Figure 4:**
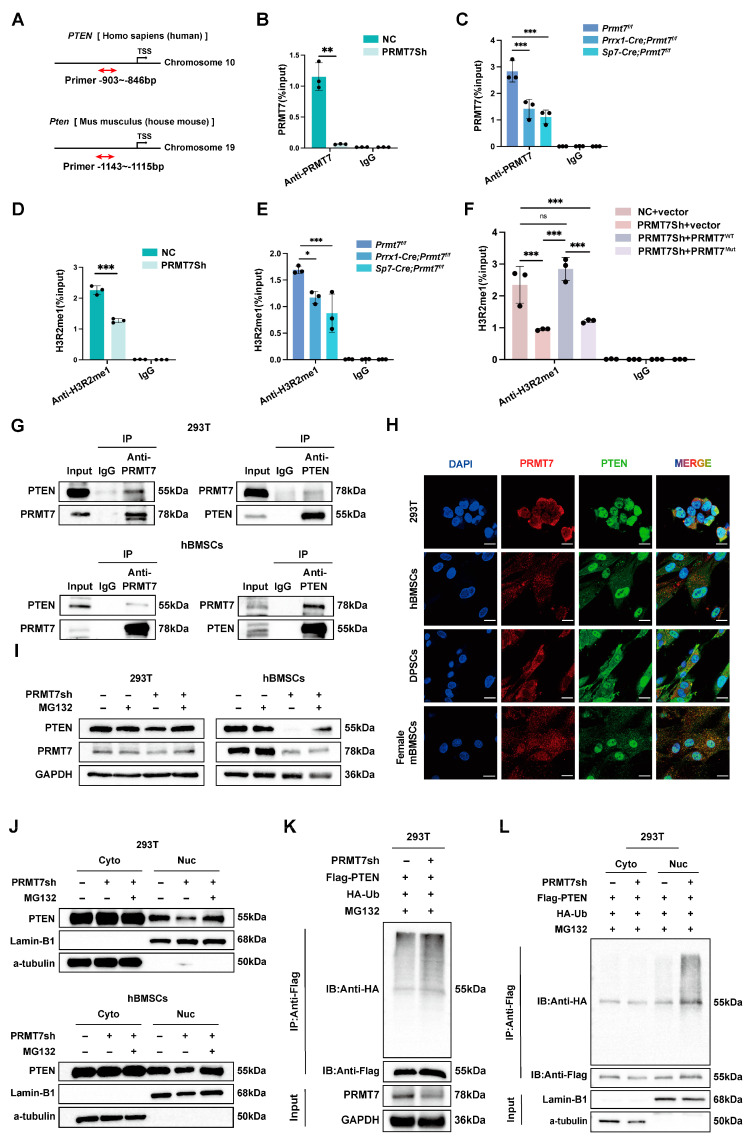
PRMT7 regulates PTEN expression and stability. (**A**) Structural schematic of human PTEN and mouse Pten genes. Red bidirectional arrows indicate regions detected by ChIP. TSS—transcription start site. (**B**) The ChIP experiments were performed using anti-PRMT7 antibodies on PRMT7sh and control hBMSCs. (**C**) The ChIP experiments were performed using anti-PRMT7 antibodies in 6-week-old female control littermates and CKO mice BMSCs. (**D**) The ChIP experiments were performed using anti-H3R2me1 antibodies on PRMT7sh and control hBMSCs. (**E**) The ChIP experiments were performed using anti-H3R2me1 antibodies in 6-week-old female control littermates and CKO mice BMSCs. (**F**) The ChIP experiments were performed using anti-H3R2me1 antibodies on hBMSCs after transfection with lentivirus carrying either an empty vector, wildtype PRMT7 (PRMT7^WT^), or enzyme-mutant PRMT7 (PRMT7^Mut^). (**G**) CoIP analysis showing the interaction of PRMT7 with PTEN in 293T and hBMSCs lysates with anti-PRMT7 and anti-PTEN antibody (pull-down with anti-PRMT7 or anti-PTEN antibody). IgG control blots confirm the specificity of the CoIP experiments. (**H**) Co-localization of PRMT7 and PTEN in 293T, hBMSCs, DPSCs, and female mBMSCs. Scale bar: 20 μm. (**I**) Representative Western blot images of PTEN in PRMT7sh and control cells. Cells were treated with or without 10 μM MG132 for 6 h prior to harvesting. (**J**) Representative Western blot images of cytoplasmic and nuclear PTEN in PRMT7sh and control cells. Cells were treated with or without 10 μM MG132 for 6 h prior to harvesting. (**K**) 293T cells were transfected with the indicated plasmid, followed by treatment with 10 μM MG132 for 6 h. Subsequently, in vivo ubiquitination assays and Western blot were conducted. (**L**) 293T cells were transfected with the plasmids, followed by treatment with 10 μM MG132 for 6 h. Subsequently, nuclear and cytoplasmic fractions were isolated for in vivo ubiquitination assays and analyzed by Western blot. This experiment was performed with two biological replicates. Unless otherwise specified, all experiments (including ChIP-qPCR and Western blot) were performed with three independent biological replicates. All data are mean ± SD. (ns—not significant, *, *p* < 0.05, **, *p* < 0.01, ***, *p* < 0.001) (One-way ANOVA).

As a protein arginine methyltransferase, PRMT7 modifies histone methylation and influences the transcriptional regulation of target genes. ChIP-qPCR results demonstrated PRMT7’s binding to the PTEN promoter, which decreased in PRMT7 knockdown cells, confirming its role (Figure 4A–C). Additionally, decreased binding of PRMT7 at the promoter regions was associated with decreased occurrence of its substrate, H3R2me1, which is a hallmark of gene activation (Figure 4D,E) [22]. Further, introducing an enzymatically inactive PRMT7 mutant in knockdown cells failed to restore H3R2me1 levels (Figure 4F), confirming that PRMT7’s role in PTEN activation depends on its enzyme activity.

Additionally, to further explore the relationship between PRMT7 and PTEN, Co-IP and immunofluorescence assays are performed to validate the interaction between PRMT7 and PTEN (Figure 4G,H; Figure S5A). Given that PRMT7 deficiency reduces both PTEN mRNA and protein levels, we aimed to determine whether the absence of PRMT7 also influences PTEN protein degradation, beyond its effect on transcription. Therefore, the proteasome inhibitor MG132 was added and the PTEN reduction induced by PRMT7 knockdown was reversed by MG132, indicating that PRMT7 affected PTEN stability through the ubiquitin-mediated protein system (Figure 4I; Figure S5B). Notably, PRMT7 knockdown selectively reduced nuclear PTEN while cytoplasmic levels remained unchanged (Figure 4J; Figure S5C). Ubiquitination assays showed increased PTEN ubiquitination following PRMT7 knockdown (Figure 4K). Next, we found that knockdown of PRMT7 only increased nuclear PTEN ubiquitination levels but not cytoplasmic PTEN (Figure 4L). These results support that knockdown of PRMT7 mediates ubiquitylation of nuclear PTEN, which is responsible for its degradation.

### 2.5. PTEN Rescued Bone Loss in Prmt7 CKO Mice

To explore PTEN’s role in PRMT7-regulated osteogenesis, we examined its function in osteogenesis. Following osteogenic induction, PTEN protein and mRNA levels increased (Figure S6A,B). ALP staining revealed significantly decreased osteogenic differentiation after PTEN deletion with siRNA (Figure S6C–E), confirming PTEN’s facilitation of MSC differentiation, consistent with prior studies on BMMSCs and DPSCs [14].

**Figure 5:**
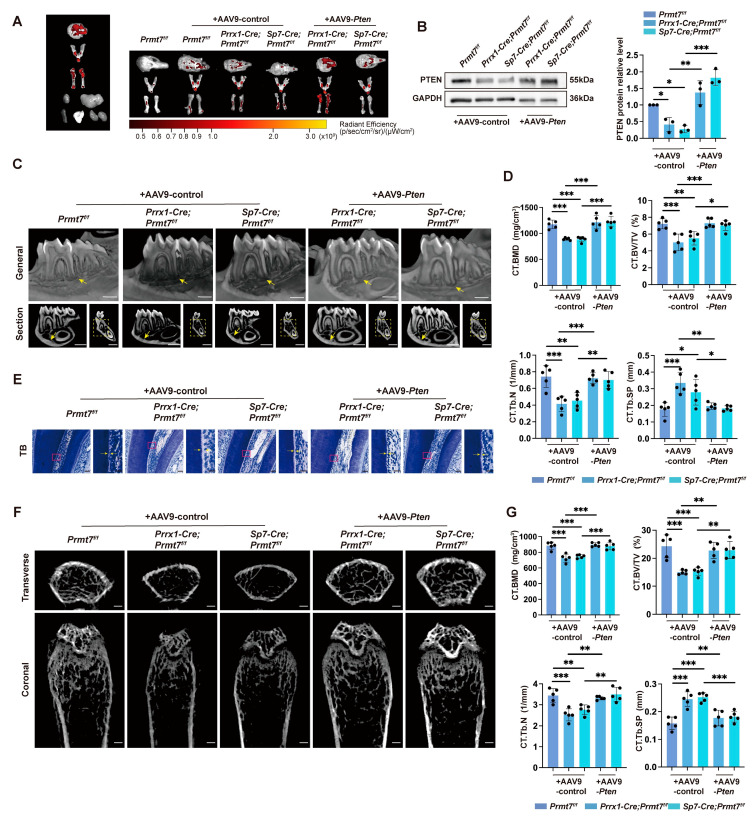
Bone loss in Prmt7 CKO mice is mitigated by PTEN. (**A**) In vivo fluorescence imaging was performed on the calvaria, mandible, femur, heart, liver, spleen, lung, kidneys (**left**), and bone tissues across all groups of mice six weeks post-lentivirus injection. Radiant efficiency is depicted using bar graphs, with measurements given in (p/sec/cm^2^/sr)/(µW/cm^2^). (**B**) Western blot analysis (**left**) of PTEN in mBMSCs, isolated from female *Prmt7^f/f^*, *Prrx1-Cre*; *Prmt7^f/f^* and *Sp7-Cre*; *Prmt7^f/f^* mice six weeks following tail vein administration of AAV9-control or AAV9-*Pten*. Quantification (**right**) of relative PTEN levels normalized to GAPDH. n = 3 biological replicates. (**C**) Micro-CT analysis of mandible from 12-week-old female *Prmt7^f/f^*, *Prrx1-Cre*; *Prmt7^f/f^*, and *Sp7-Cre*; *Prmt7^f/f^* mice after 6-week of AAV9-control or AAV9-*Pten* injection. The **upper** panel: 3D reconstructed sectional view of the mandible; the **lower left** panel: mesiodistal sectional view of the first molar; the **lower right** panel: coronal sectional view of the first molar. The yellow arrow at the **upper** panel points to the bone around the apical area of the distal root of the first molar; the yellow arrow at the **lower left** panel points to the bone wall at the **lower border** of the mandibular body; the yellow dashed box at the **lower right** panel indicates the bone between the furcation of the first molar and the mandibular canal. Scale bar: 1 mm. (**D**) Bone parameters quantitative analysis of mandible, including BMD, BV/TV, Tb. N, and Tb. Sp, obtained in C. Each group consists of 5 mice, with a total of 25 mice. (**E**) Toluidine blue staining of the dental tissues at the alveolar crest of the distal root of the first molar from 12-week-old female *Prmt7^f/f^*, *Prrx1-Cre*; *Prmt7^f/f^*, and *Sp7-Cre*; *Prmt7^f/f^* mice after 6 weeks of AAV9-control or AAV9-*Pten* injection. The yellow arrow in the right points to the predentin, which is an enlarged view of the area in the pink box in the left. Scale bars: 50 μm (**left**) and 20 μm (**right**). (**F**) Micro-CT analysis of femurs from 12-week-old female *Prmt7^f/f^*, *Prrx1-Cre*; *Prmt7^f/f^*, and *Sp7-Cre*; *Prmt7^f/f^* mice after 6 weeks of AAV9-control or AAV9-*Pten* injection. The **upper** panel shows the cross-sectional view of the metaphysis; the **lower** panel shows the coronal view of the metaphysis. Scale bar: 500 μm. (**G**) Quantitative analysis of bone parameters at the femoral metaphysis growth plate, including BMD, BV/TV, Tb. N, and Tb. Sp, obtained in F. Each group consists of 5 mice, with a total of 25 mice. All data are mean ± SD. (*, *p* < 0.05, **, *p* < 0.01, ***, *p* < 0.001) (One-way ANOVA).

To determine if PTEN could reverse Prmt7-induced bone loss in vivo, we used adeno-associated virus serotype 9 (AAV9) for systemic delivery of PTEN. After 6 weeks of injection in female Prmt7-CKO and control mice, AAV9 efficiently targeted major organs and bones (Figure 5A,B). H&E staining showed no significant effects on the liver (Figure S6F). Micro-CT analysis demonstrated that PTEN reversed bone loss from PRMT7 deletion in the mandible and femur. Bone parameters indicated that BMD, BV/TV, and Tb. N decreased in CKO mice but increased in AAV9-Pten groups, while Tb. Sp showed an opposite trend (Figure 5C,D,F,G). Predentin width in AAV-Pten groups was also greater, indicating normal biomineralization (Figure 5E). These results suggest PTEN can reverse bone loss in Prmt7-deficient female mice.

In vitro, we extracted BMSCs and added Pten-overexpression or control plasmids. ALP staining confirmed PTEN as an intermediate target between PRMT7 and osteogenesis, enhancing osteogenic differentiation reduced by Prmt7 deletion (Figure S6G). RUNX2 levels further supported these findings (Figure S6H,I). Thus, PTEN is essential for bone formation in Prmt7-deficient female mice.

## 3. Discussion

Our study reveals that PRMT7 specifically enhances bone formation and regeneration in the axial and appendicular bones of female mice, with no impact on males, through the construction of CKO mouse models. Most importantly, we discovered that PRMT7 transcriptionally activates PTEN and regulates its nuclear stabilization, a novel finding not reported previously. This identifies PRMT7 as a key regulator of PTEN, opening new avenues for developing therapeutic strategies aimed at enhancing bone regeneration and treating bone-related disorders, such as osteopenia and osteoporosis.

Initially, PRMT7 was mistakenly identified as an enzyme that catalyzes the formation of symmetrical dimethylation products due to contamination with PRMT5 [23,24]. For instance, a previous study reported that PRMT7 catalyzed H3R2me2s to enhance binding with WDR5 in euchromatin [25]. However, current research clearly demonstrates that PRMT7 can only catalyze MMA [26], and the observed alteration of SDMA may be due to crosstalk with other PRMTs via secondary effects, which has not been fully confirmed [27]. Given PRMT7’s ability to produce only MMA, we focused on its direct effect on H3R2. PRMT7 has been reported to be responsible for H3R2me1 at the promoter of *Rap1a*, affecting monocyte characteristics [21]. H3R2me1 is associated with gene transcriptional activation, similar to H3R2me2s but opposite to H3R2me2a [28,29,30]. We confirmed that H3R2me1 serves as a substrate of PRMT7 in BMSCs, regulating osteogenic differentiation. The loss of PRMT7 decreased the global expression level of H3R2me1 in MSCs and reduced its presence at the promoter of the target gene *PTEN*, thereby impacting its transcription. Notably, a PRMT7 enzymatic dead mutant could not rescue the reduction in global H3R2me1 levels and its presence at the *PTEN* promoter caused by PRMT7 knockdown. Consequently, PRMT7 can be targeted in cell-mediated regenerative medicine to regulate osteogenic differentiation, influencing bone formation and regeneration.

The regulation of PTEN is governed by a multitude of molecular mechanisms. Several studies have elucidated the regulatory influence of the PRMT family on PTEN via distinct mechanisms. For instance, PRMT5 represses PTEN expression at the transcriptional level in glioblastoma neurospheres (GBMNSs) by binding to its promoter [31]. Additionally, PTEN undergoes ADMA at R159 through its interaction with PRMT6, which affects mRNA alternative splicing [18]. Our current study introduces novel insights into the PRMT–PTEN axis. Unlike cytoplasmic PTEN, which primarily functions in a phosphatase-dependent manner, nuclear PTEN performs various roles independent of its phosphatase activity [32]. For example, the loss of nuclear PTEN can lead to more aggressive tumors by regulating genomic stability and the cell cycle [33]. Prior research has demonstrated that the nuclear export of PTEN, rather than the inhibition of its phosphatase activity, compromises the activation of the anaphase promoting complex/cyclosome (APC)-CDC20like protein 1 (CDH1) complex, consequently reducing its tumor-suppressive function [34]. Given the distinct roles of nuclear versus cytoplasmic PTEN and the importance of nuclear PTEN, it is imperative to investigate the regulatory mechanisms and functional pathways governing these differences. Emerging research highlights that the knockdown of NEDD4-1 and WWP2, two oncogenic ubiquitin ligases, selectively augments cytoplasmic PTEN levels without affecting nuclear PTEN [35,36]. Conversely, the E3 ligase FBXO22 specifically ubiquitinates nuclear PTEN, impacting its stability [37]. There is also ongoing debate regarding the relative stability of nuclear versus cytoplasmic PTEN, potentially due to differences in endogenous versus exogenous sources of PTEN protein. Overexpression of exogenous PTEN can lead to protein instability and challenges in nuclear localization [37,38]. Our research demonstrates that PRMT7 acts as a novel stabilizer of nuclear PTEN, enhancing its stability without affecting cytoplasmic PTEN, thus promoting osteogenesis. Given that PRMT7 lacks E3 ubiquitin ligase activity, we propose that PRMT7 may function as an adaptor protein, facilitating the recruitment of an E3 ubiquitin ligase to PTEN or competitively bind with PTEN’s deubiquitinase to mediate its ubiquitination. This hypothesis necessitates further exploration. In a word, our results provide new insights into the relative stability of nuclear PTEN and expand the functional role of nuclear PTEN in stem cell biology.

One intriguing aspect of our study is that PRMT7 promotes osteogenesis exclusively in female mice, without affecting male mice. Upon PRMT7 knockdown, PTEN expression levels decreased solely in female mice, with no significant change in males. This suggests PTEN may be a crucial mediator of the sex-specific bone phenotype driven by PRMT7. Numerous studies highlight the pervasive nature of sex differences across species, tissues, and biological processes [39,40]. These differences may be gene activity-related and induced. While gene expression on sex chromosomes is a major source, the sex-biased expression of autosomal genes, often tissue-specific, is also noteworthy [41]. PTEN, located on chromosome 10 in humans and chromosome 19 in mice, exhibits sex-dependent tissue specificity, encompassing adipose, central nervous system, thyroid gland, and hepatic tissue [42,43,44,45]. For example, a high-fat diet raises AEBP1 levels in white adipose tissue and reduces PTEN expression in female mice, while male mice show no significant changes in AEBP1 or PTEN [42]. These differences are primarily linked to sex hormone levels, with several studies indicating a close correlation between hormone/hormone receptor expression and PTEN levels [46,47]. In endometrial cancer, estrogen binds to ER, increasing miR-200c levels and inhibiting PTEN and PTENP1, which activates the PI3K-AKT pathway [46]. Although our RNA-seq results showed no significant differences in estrogen and receptor levels between female CKO and control mice, the higher estrogen levels in females compared to males may explain the PTEN alterations observed exclusively in female mice, warranting further investigation [47]. Taken together, our study highlights gender-specific differences in PTEN expression in bone tissue, reinforcing evidence of its modulation by sex.

## 4. Materials and Methods

### 4.1. Experimental Animals

C57BL/6J mice were used for all experiments, maintained in a specific pathogen-free (SPF) environment with a 12 h light/dark cycle. *Prmt7* conditional knockout (*Prmt7^f/f^*) mice were generated by inserting LoxP sites flanking exons 3 and 4. *Prmt7^f/f^* mice were crossed with *Prrx1-cre* and *Sp7-cre* mice to generate *Prrx1-cre; Prmt7^f/f^*, and *Sp7-cre; Prmt7^f/f^* lines. Genotyping was performed, and related primers are listed in Table S1. The experimental unit for all experiments was a single mouse, allowing for independent and detailed analysis of the phenotype of each mouse. The criteria for selecting mice that meet the experimental requirements are primarily based on sex, genotype, age, and overall health status. Since our study requires differentiation based on sex (male and female), different genotypes (*Prrx1-cre; Prmt7^f/f^*, *Sp7-cre; Prmt7^f/f^*, and *Prmt7^f/f^*), and various age groups (E18.5, P0, 6 weeks), any mice that do not meet the required sex or genotype, have diseases, are of an inappropriate age, or display abnormal behavior that may not align with the research phenotype, will be excluded. During our research, we did not encounter any mice exhibiting abnormal behavior or health issues. The surplus mice were used for breeding or for preserving the strain. A total of 4 pregnant genetically modified dams were used to harvest E18.5 embryos at 18 days post-plug detection. Developmental phenotyping included 6 P0 neonates (3 strains × 2 sexes). For postnatal analyses, 170 six-week-old mice (3 strains × 2 sexes) were allocated to morphological and molecular assays: CT imaging utilized 5 mice per group, while alkaline phosphatase (ALP) staining, qRT-PCR, and Western blot were performed with 3 biological replicates per group. AAV intervention studies employed 41 twelve-week-old mice, with CT imaging (n = 5/group) and downstream analyses (n = 3 biological replicates). Sex-matched wild-type controls (3 males, 3 females) were included for baseline comparisons. In the experiment, mice were genotyped and labelled, followed by random assignment of experimental units to the control group and treatment group using Excel randomization. To minimize the impact of confounding factors, such as treatment and measurement order, a fully randomized treatment sequence was implemented, and animals were randomly allocated between different cages. All experimental procedures were approved by the Peking University Health Science Center Animal Care and Use Committee (LA2022005).

### 4.2. Defect Models

We used an equal number of both female and male mice from each strain for the study. For mandibular defects, mice were anesthetized with isoflurane (induction: 5%, maintenance: 1.5–2%). After confirming anesthesia depth by absence of pedal reflex, mice were positioned laterally. The unilateral right mandibular region was shaved and disinfected with iodophor. A 3 mm skin incision parallel to the mandibular border was made below the tragus-oral commissure line. Blunt dissection through musculature exposed the mandibular surface while preserving neurovascular structures. A 1.5 mm spherical drill was used to create full-thickness cortical defects (confirmed by penetration loss). The wound was irrigated with saline, followed by layered closure. Postoperative care included the following: (1) continuous monitoring until recovery of righting reflex; (2) hydrogel diet supplementation for 72 h; (3) defect harvest at 8 weeks. For tibial defect, anesthetized mice (protocol as above) were positioned supine. The proximal tibial metaphysis (anteromedial surface) was exposed through a 3 mm incision. Subperiosteal dissection created a drilling window. A 1 mm spherical drill generated transcortical defects. Surgical closure and postoperative monitoring followed the mandibular protocol, with defect harvest at 10 days.

### 4.3. Micro-CT and Quantitative Analysis

Bone samples were fixed in 4% paraformaldehyde. Micro-CT scanning was performed using a Bruker SkyScan 1276 system (Bruker, Kontich, Belgium). Scanning parameters were configured as follows: X-ray source voltage 80 kV, current 200 μA, 0.25 mm aluminum filtration, and spatial resolutions of 10 μm/pixel (mandible) or 6 μm/pixel (femur), with an image resolution of 2048 × 2048 pixels. 2D images were acquired using DataViewer (v1.5.2.4; Bruker), followed by 3D reconstruction with CTVol v2.0 (Bruker). Morphometric parameters of bone tissue were quantified using CTAn v1.2 (Bruker). For all CT analyses, the sample size in each group was n = 5, which is generally sufficient to detect significant differences between the experimental and control groups. This sample size helped us quickly assess the feasibility and effectiveness of the experiment in exploratory studies.

### 4.4. Whole-Body Skeleton Staining

Specimens were dissected using forceps to remove integument and visceral tissues, followed by immediate fixation in 95% ethanol for 24 h. Fixed specimens underwent cartilage staining in an alcian blue solution (pH 2.5) for 72 h, with subsequent destaining in 95% ethanol for an equivalent duration. Tissue clarification was achieved through immersion in 2% potassium hydroxide solution for 24 h. Osseous structures were then differentially stained with 0.015% alizarin red S solution for 24 h. Final preservation was maintained in a stabilizing solution containing 20% glycerol and 1% potassium hydroxide (KOH).

### 4.5. Histological Staining

Bone and liver tissues were fixed in 10% paraformaldehyde and decalcified in 10% EDTA-2Na for 2 weeks (bone tissues). The samples were sequentially immersed in a graded ethanol series for dehydration (70%, 80%, 95%, 100%), followed by clearing with xylene through two cycles (1 h each). Subsequently, they were embedded in paraffin and sectioned into 4–6 μm thick tissue slices. Finally, deparaffinization was performed using xylene, followed by rehydration through a descending ethanol series until the sections were ready for aqueous staining. Then the samples stained with H&E, Masson’s trichrome, and toluidine blue.

### 4.6. Immunofluorescence

Bone tissue samples from mice were fixed in 10% neutral formalin for at least 48 h, followed by decalcification in 10% EDTA-2Na for two weeks. After decalcification, tissues were washed three times with PBS, then incubated in 20% sucrose for settling, followed by 30% sucrose at 4 °C overnight. The tissue was considered fully dehydrated when it settled at the bottom of the tube. Tissue was embedded in OCT medium, positioned in the embedding mold, frozen, and sectioned using a cryostat. Sections were washed three times with PBS, then permeabilized with 0.5% Triton X-100 (Merck KGaA, Darmstadt, Germany) for 10 min. After additional PBS washes, sections were blocked for 1 h in 1% BSA. After blocking, primary antibodies (100 µL per slide) were applied overnight at 4 °C. The slides were then washed and incubated with fluorescence-conjugated secondary antibodies for 2 h, protected from light. Finally, sections were stained with DAPI for nuclear visualization and imaged using a confocal microscope (Olympus FV3000, Olympus Corporation, Tokyo, Japan).

### 4.7. Cell Extraction, Culture, and In Vitro Assays

Bone marrow stem cells (BMSCs) were flushed from mouse femurs and cultured in α-MEM with 10% fetal bovine serum (FBS) and 1% penicillin/streptomycin (P/S). For osteogenic differentiation, cells were treated with induction media containing 100 nM dexamethasone, 0.2 mM vitamin C, and 10 mM β-glycerophosphate. Alkaline phosphatase (ALP) staining and quantification were performed after 7 days.

### 4.8. Lentiviral Transduction and Plasmid, siRNA Transfection

Cells were transduced with lentiviral vectors expressing PRMT7sh, PRMT7, or controls, followed by puromycin selection. Efficiency was confirmed by fluorescence microscopy, Western blot, and qPCR. Transfections with plasmids and siRNA were performed using Lipofectamine 3000, and cells were harvested 48 h later.

### 4.9. Protein Extraction and Western Blot

Cells were lysed in RIPA buffer, and protein concentrations were determined with a BCA assay. Equal amounts of protein (20 μg) were separated by SDS-PAGE using 80 V constant voltage in stacking gels and 120 V in separating gels. Proteins were subsequently transferred to PVDF membranes under constant voltage (100 V) for 45 min (Bio-Rad, Hercules, CA, USA). Membranes were blocked with 5% skim milk prepared in TBST buffer at room temperature for 1 h to prevent nonspecific binding. Primary antibody incubation was performed overnight at 4 °C, followed by three TBST washes to remove unbound antibodies. Membranes were then incubated with HRP-conjugated secondary antibodies at room temperature for 1 h. After three additional TBST washes, protein bands were visualized using enhanced chemiluminescence (ECL) reagent and captured with a digital imaging system. Band intensity quantification was performed using ImageJ software (2.0.0-rc-69/1.52p). Nuclear and cytoplasmic fractions were extracted using the NE-PER™ kit. Cells were harvested, lysed, and proteins were stored at −80 °C. The remaining procedures are the same as above.

### 4.10. RNA Extraction and qRT-PCR

Total RNA was isolated using TRIzol and quantified with a NanoDrop spectrophotometer (Thermo Fisher Scientific, Wilmington, DE, USA). cDNA was synthesized from 1 µg RNA, and qPCR was performed using SYBR Green PCR master mix using a real-time PCR system (Applied Biosystems, Foster City, CA, USA). Expression levels were normalized to *GAPDH*.

### 4.11. RNA Sequencing and Analysis

RNA from female *Prmt7^f/f^*, *Prrx1-Cre*; *Prmt7^f/f^*, and *Sp7-Cre*; *Prmt7^f/f^* BMSCs was extracted and sequenced on an Illumina platform. Differentially expressed genes (DEGs) were identified using DESeq2 with *p* < 0.05 and fold change > 1.5. GO and KEGG pathway enrichment was performed using clusterProfiler v4.0.0.

### 4.12. ChIP-qPCR

Cells were fixed with 1% formaldehyde for crosslinking, and the reaction was quenched with 0.125 M glycine. Chromatin was fragmented into 200–500 bp fragments using sonication (Covaris M220 Focused-ultrasonicator, Woburn, MA, USA). Immunoprecipitation was performed with target-specific antibodies and Protein A/G magnetic beads (MCE). After washing, the precipitated complexes were reverse-crosslinked at 65 °C and digested overnight with proteinase K. Purified DNA was analyzed by qPCR using specific primers (Table S1). Data were normalized to input DNA and quantified using the ΔΔCt method.

### 4.13. Immunoprecipitation and In Vivo Ubiquitination Assay

The cells were lysed in IP lysis buffer (Epizyme Biotech, Shanghai, China) containing protease inhibitors. After preclearing with protein A/G magnetic beads, the lysates were incubated with IP-grade antibodies (Anti-PRMT7 or Anti-PTEN) overnight at 4 °C, followed by capture with protein A/G magnetic beads (4–6 h). The immunocomplexes were washed and eluted in SDS sample buffer. Eluted proteins were separated by SDS-PAGE and analyzed via Western blot using corresponding antibodies.

To detect PTEN ubiquitination, HEK293T and PRMT7sh cells were transfected with plasmids encoding HA-Ub and FLAG-PTEN. After 48 h, cells were treated with 10 μM MG132 (MCE) for 6 h to inhibit proteasomal degradation. Cells were lysed in IP buffer containing protease/phosphatase inhibitors or fractionated using the NE-PER™ kit. Lysates were clarified by centrifugation (14,000 rpm, 15 min, 4 °C), and supernatants were subjected to immunoprecipitation with anti-FLAG magnetic beads overnight at 4 °C. Beads were washed with RIPA buffer, and bound proteins were eluted in SDS-PAGE loading buffer by boiling. Ubiquitinated PTEN was analyzed via Western blot using anti-HA and anti-FLAG antibodies.

### 4.14. Co-Localization Assay

The cells were fixed with 4% paraformaldehyde for 15 min, followed by permeabilization with 0.1% Triton X-100 to facilitate antibody penetration. After blocking with 5% normal goat serum to minimize nonspecific binding, double immunofluorescence staining was performed using primary antibodies targeting PRMT7 and PTEN and then incubated at 4 °C overnight. The samples were then treated with fluorophore-conjugated secondary antibodies at room temperature for 1 h. Unbound antibodies were removed by three PBS washes (5 min each). Fluorescent images were captured using an Olympus FV3000 fluorescence microscope.

### 4.15. Intravenous Injection and In Vivo Imaging

Six-week-old female *Prmt7^f/f^*, *Prrx1-Cre*; *Prmt7^f/f^*, and *Sp7-Cre*; *Prmt7^f/f^* mice were utilized for tail vein injection. Prior to injection, mice were gently restrained to expose the tail vein. AAV-*Pten* or AAV-control (titer: 2.2 × 10^12^ TU/mL, 100 μL per injection) was administered via insulin syringe, ensuring precise insertion into the vein to minimize vascular damage or leakage. Post-injection monitoring was performed to detect adverse reactions. Injections were administered biweekly (total two doses), and samples were collected 6 weeks after the initial injection.

### 4.16. Statistical Analysis

Data were analyzed using GraphPad Prism 9. Continuous data were expressed as mean ± SD, and group comparisons were made using *t*-tests or ANOVA. A *p*-value < 0.05 was considered significant. Normality was assessed using the Shapiro–Wilk test.

## Data Availability

The study data are available upon request from the corresponding author.

## Supplementary Materials

The following supporting information can be downloaded at: https://www.mdpi.com/article/10.3390/ijms26072981/s1.
