# Supplementary material for: PRMT7-Mediated PTEN Activation Enhances Bone Regeneration in Female Mice
Source: Int J Mol Sci. 2025 Mar 25;26(7):2981. doi: 10.3390/ijms26072981 (PMC11988880; doi:10.3390/ijms26072981)
Supplement: Supplementary file 1 [file ijms-26-02981-s001.zip › ijms-3511470-supplementary.pdf]

Supplementary Material

PRMT7-mediated PTEN activation enhances bone regeneration in female mice

Authors: Yingfei Zhang[1,2], Jia Qing[1,2], Yang Li[1,2], Xin Gao[1,2], Dazhuang Lu[1,2], Yiyang Wang[1,2], Lanxin Gu[1,2], Hui Zhang[1,2], Zechuan Li[1,2], Xu Wang[1,2], Yongsheng Zhou[1,2*], Ping Zhang[1,2*]

Affiliations:

1 Peking University Hospital of Stomatology, Department of Prosthodontics, 100081 Beijing, China.

2 Peking University Hospital of Stomatology National Clinical Research Center for Oral Diseases, 100081 Beijing, China.

*Correspondence: zhangping332@bjmu.edu.cn, kqzhouysh@hsc.pku.edu.cn.

Table of contents: Page Number

Figure S1	2
Figure S2	5
Figure S3	7
Figure S4	9
Figure S5	11
Figure S6	12
Table S1	13

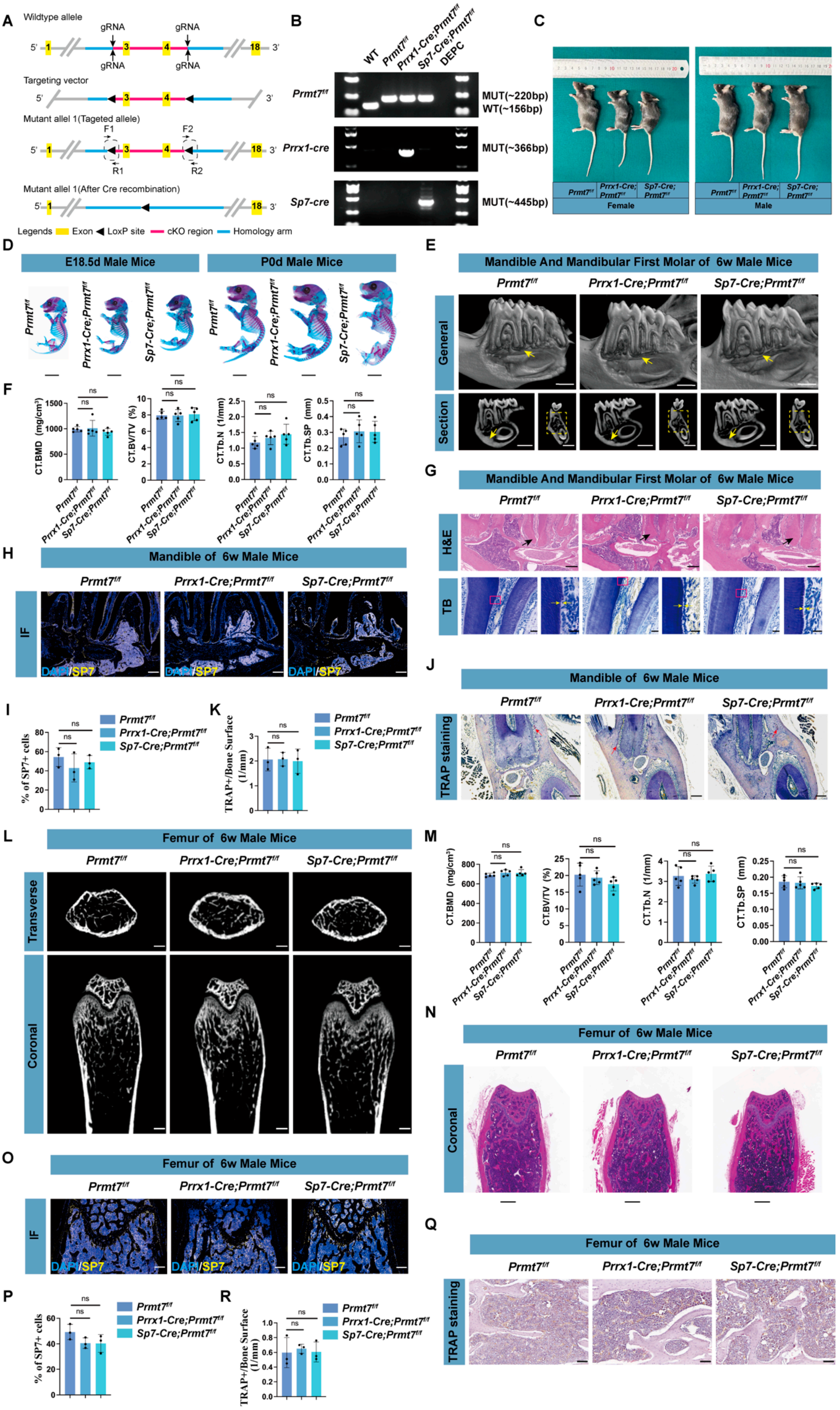
A
Wildtype allele
gRNA
gRNA
gRNA
gRNA
Targeting vector
Mutant allel 1(Tageted allele)
F1
F2
R1
R2
Mutant allel 1(After Cre recombination)
Legends Exon LoxP site cKO region Homology arm
B
WT
Prmt7f/f
Prrx1-Cre;Prmt7f/f
Sp7-Cre;Prmt7f/f
DEPC
Prmt7f/f
Prrx1-cre
Sp7-cre
MUT(~220bp)
WT(~156bp)
MUT(~366bp)
MUT(~445bp)
C
Female
Male
D
E18.5d Male Mice
P0d Male Mice
E
Mandible And Mandibular First Molar of 6w Male Mice
General
Section
F
CT.BMD (mg/cm³)
CT.BV/TV (%)
CT.Tb.N (1/mm)
CT.Tb.SP (mm)
ns
G
Mandible And Mandibular First Molar of 6w Male Mice
H&E
TB
H
Mandible of 6w Male Mice
IF
DAPI/SP7
I
% of SP7+ cells
K
TRAP+/Bone Surface (1/mm)
J
Mandible of 6w Male Mice
TRAP staining
L
Femur of 6w Male Mice
Transverse
Coronal
M
N
Femur of 6w Male Mice
Coronal
O
Femur of 6w Male Mice
IF
DAPI/SP7
Q
Femur of 6w Male Mice
TRAP staining
P
% of SP7+ cells
R
TRAP+/Bone Surface (1/mm)
Prmt7f/f
Prrx1-Cre;Prmt7f/f
Sp7-Cre;Prmt7f/f

Figure S1: The deficiency of PRMT7 has no effect on bone and dental structures in male mice.

A. Construction strategy diagram of control (*Prmt7$^{f/f}$*) and CKO mice (*Prrx1-cre; Prmt7$^{f/f}$* and *Sp7-cre; Prmt7$^{f/f}$*).

B. Genotype identification of control (*Prmt7$^{f/f}$*) and CKO mice (*Prrx1-cre; Prmt7$^{f/f}$* and *Sp7-cre; Prmt7$^{f/f}$*).

C. Overall physical size of 6-week-old female and male control and CKO mice.

D. Alcian Blue and Alizarin Red staining of E18.5 and P0 male control and CKO mice. Scale bar: 5mm.

E. Micro-CT analysis of the mandible in 6-week-old male control and CKO mice. The upper panel: 3D reconstructed sectional view of the mandible; The lower left panel: mesiodistal sectional view of the first molar; The lower right panel: coronal sectional view of the first molar. The yellow arrow at the upper panel points to the bone around the apical area of the distal root of the first molar; the yellow arrow at the lower left panel points to the bone wall at the lower border of the mandibular body; the yellow dashed box at the lower right panel indicates the bone between the furcation of the first molar and the mandibular canal. Scale bar: 1mm.

F. Bone parameters quantitative analysis of the mandible in 6-week-old male control and CKO mice, including BMD, BV/TV, Tb. N, and Tb. Sp, obtained in E.

G. H&E staining (The upper panel) of the periapical bone in the mandible of 6-week-old male control and CKO mice. The black arrow indicates the cementum in the apical area of the distal root of the first molar, while the yellow arrow points to the bone wall at the lower border of the mandible. Toluidine Blue staining (the lower panel) of the dental tissues at the alveolar crest of the distal root of the first molar. The yellow arrow in the lower right points to the predentin, which is an enlarged view of the area within the pink box in the lower left. Scale bars: 200 μm, 50 μm and 20 μm.

H. Immunofluorescence staining of SP7 in the mandible of 6-week-old male *Prmt7$^{f/f}$*, *Prrx1-Cre; Prmt7$^{f/f}$* and *Sp7-Cre; Prmt7$^{f/f}$* mice. Scale bar: 200 μm.

I. Quantitative analyses of the ratio of $SP7^+$ cells to total cells in the mandible of 6-

week-old male *Prmt7*$^{f/f}$, *Prrx1-Cre; Prmt7*$^{f/f}$ and *Sp7-Cre; Prmt7*$^{f/f}$ mice.

J. Tartrate-resistant acid phosphatase (TRAP) staining was performed on the first molar region of the mandible in male CKO mice and their control group. Scale bar: 200 μm.

K. Quantitative analysis of the number of TRAP^{+} osteoclasts / bone surface (1/mm) in the mandible of male CKO mice and their control group.

L. Micro-CT analysis of femurs from 6-week-old male control littermates and CKO mice. The upper panel shows the cross-sectional view of the metaphysis; The lower panel shows the coronal view of the metaphysis. Scale bar: 500 μm.

M. Quantitative analysis of bone parameters at the femoral metaphysis growth plate, including BMD, BV/TV, Tb. N, and Tb. Sp, obtained in L.

N. H&E staining of the coronal section of the femur in 6-week-old male control littermates and CKO mice. Scale bar: 500 μm.

O. Immunofluorescence staining of SP7 in the femur of 6-week-old male *Prmt7*$^{f/f}$, *Prrx1-Cre; Prmt7*$^{f/f}$ and *Sp7-Cre; Prmt7*$^{f/f}$ mice. Scale bar: 200 μm.

P. Quantitative analyses of the ratio of SP7^{+} cells to total cells in the femur of 6-week-old male *Prmt7*$^{f/f}$, *Prrx1-Cre; Prmt7*$^{f/f}$ and *Sp7-Cre; Prmt7*$^{f/f}$ mice.

Q. Tartrate-resistant acid phosphatase (TRAP) staining was performed on the femur of 6-week-old male CKO mice and their control group. Scale bar: 100 μm.

R. Quantitative analysis of the number of TRAP^{+} osteoclasts / bone surface (1/mm) in the femur of 6-week-old male CKO mice and their control group.

Data were expressed as mean ± standard deviation (SD) and analyzed by one-way ANOVA. ns, no significant; *p < 0.05; **p < 0.01; ***p < 0.001. For all CT analyses, the sample size in each group was n=5. The sample size for immunofluorescence and TRAP quantification was n = 3 mice per genotype.

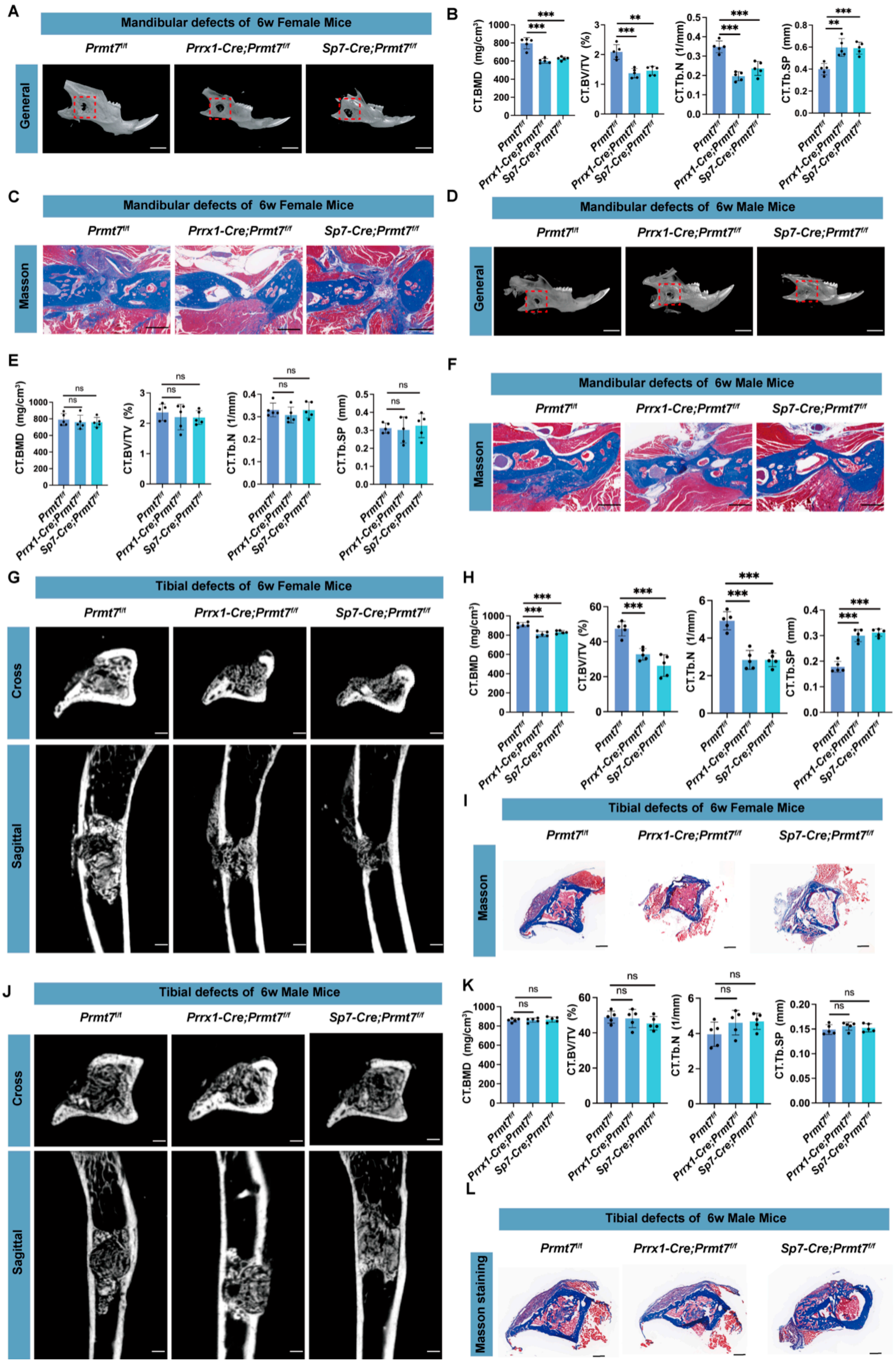

Figure S2: The deficiency of PRMT7 affects bone regeneration in female mice.

A. Micro-CT analysis of the mandibular defect model in female control (*Prmt7*$^{f/f}$) and

CKO mice (*Prrx1-cre; Prmt7*$^{f/f}$ and *Sp7-cre; Prmt7*$^{f/f}$). The red dashed box indicates the defect area. scale bar: 2 mm.

B. Quantitative analysis of bone parameters in the defect region of the mandibular defect model obtained in A.

C. Cross-sectional Masson's trichrome staining of the obtained mandibular defect models in female control and CKO mice. Scale bar: 500 μm.

D. Micro-CT analysis of the mandibular defect model in male control and CKO mice. The red dashed box indicates the defect area. scale bar: 2 mm.

E. Quantitative analysis of bone parameters in the defect region of the mandibular defect model obtained in D.

F. Cross-sectional Masson's trichrome staining of the obtained mandibular defect models in male control and CKO mice. Scale bar: 500 μm.

G. Micro-CT analysis of tibial defect modeling in 6-week-old female control littermates and CKO mice, with samples collected 10 days post-operation. The upper panel shows the cross-sectional view of the defect site; the lower panel shows the sagittal view of the tibia sample. Scale bar: 500 μm.

H. Quantitative analysis of bone parameters in the defect region of the tibial defect model obtained in G.

I. Cross-sectional Masson's trichrome staining of the obtained tibial defect models in 6-week-old female mice. Scale bar: 500 μm.

J. Micro-CT analysis of tibial defect modeling in 6-week-old male control littermates and CKO mice, with samples collected 10 days post-operation. The upper panel shows the cross-sectional view of the defect site; the lower panel shows the sagittal view of the tibia sample. Scale bar: 500 μm.

K. Quantitative analysis of bone parameters in the defect region of the tibial defect model obtained in J.

L. Cross-sectional Masson's trichrome staining of the obtained tibial defect models in 6-week-old male mice. Scale bar: 500 μm.

Data were expressed as mean ± standard deviation (SD) and analyzed by one-way ANOVA. ns, no significant; *p < 0.05; **p < 0.01; ***p < 0.001. For all CT analyses, the sample size in each group was n=5.

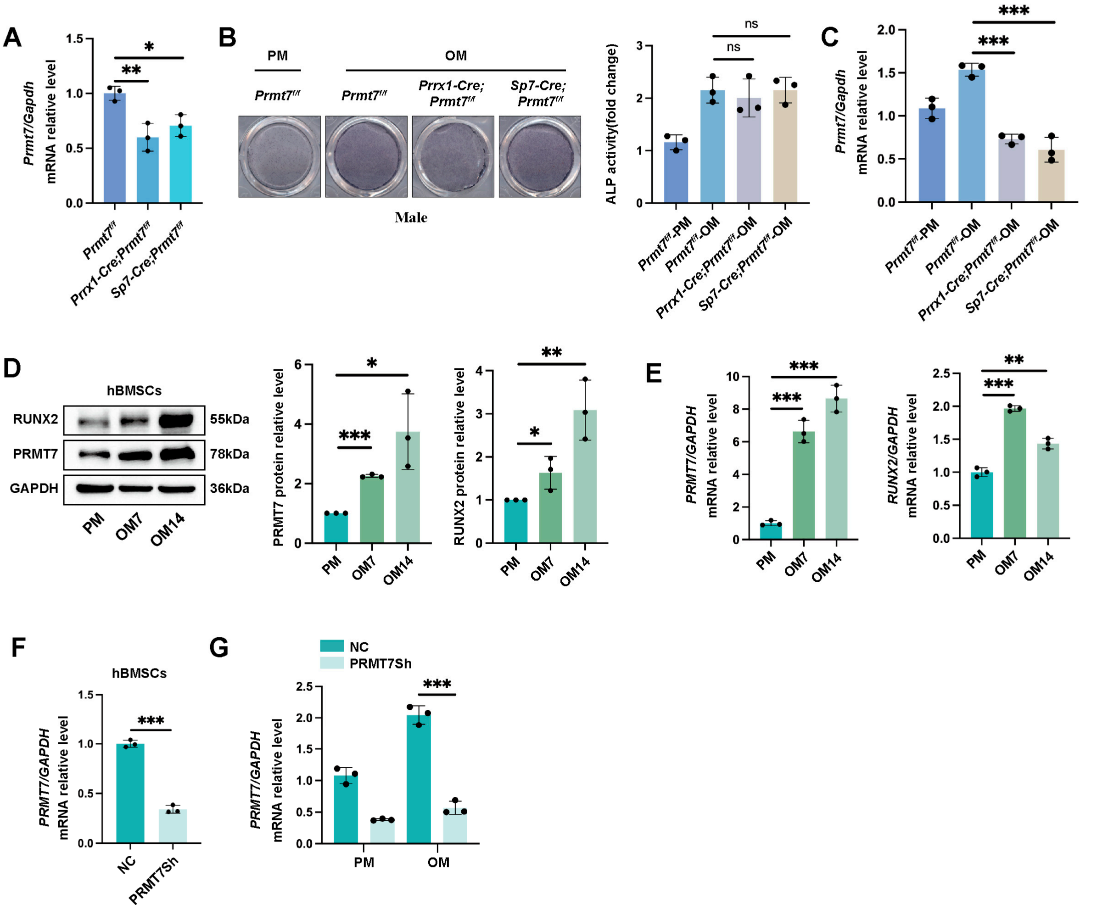

Figure S3: PRMT7 regulates osteogenic differentiation via its methyltransferase activity, related to Figure2.

A. qRT-PCR results of *Prmt7* mRNA levels in 6-week-old female control littermates (*Prmt7$^{f/f}$*) and CKO mice (*Prrx1-cre; Prmt7$^{f/f}$* and *Sp7-cre; Prmt7$^{f/f}$*) BMSCs.

B. Staining and quantification of ALP in 6-week-old male control littermates and CKO mice BMSCs after 7 days of osteogenic induction.

C. qRT-PCR results of *Prmt7* in 6-week-old female control littermates and CKO mice BMSCs after 7 days of osteogenic induction.

D. Representative Western blot images (left) of PRMT7 and RUNX2 in hBMSCs after 7 days and 14 days of osteogenic induction. Quantification (right) of relative PRMT7 and RUNX2 levels normalized to GAPDH.

E. qRT-PCR results of *PRMT7* and *RUNX2* in hBMSCs after 7 days and 14 days of osteogenic induction.

F. qRT-PCR results of *PRMT7* in hBMSCs after transfection with PRMT7sh and control lentivirus respectively.

G. qRT-PCR results of *PRMT7* in PRMT7sh and control hBMSCs after 7 days of osteogenic induction.

All data are mean ± SD, n = 3 biological replicates. (*, P <0.05, **, P <0.01, ***, P <0.001) (Independent samples t-test or One-way ANOVA).

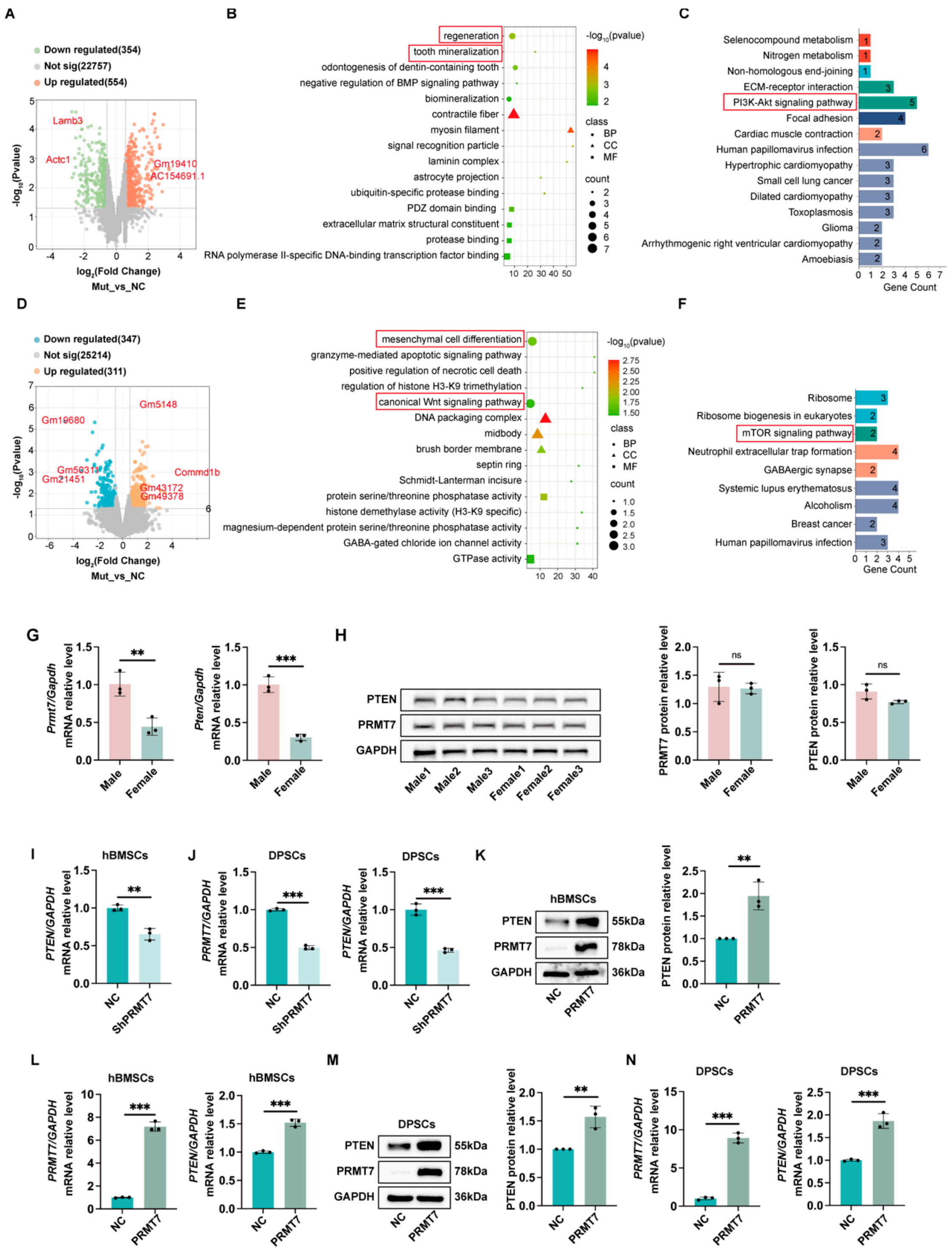

Figure S4. PRMT7 induces PTEN activation in female mice, related to Figure3.

A. Volcano plot showing the comparison of differentially expressed genes between female *Prmt7*$^{f/f}$ and *Prrx1-Cre; Prmt7*$^{f/f}$ groups, highlighting significantly upregulated and downregulated genes.

B. KEGG pathway analysis identifying enriched pathways in the differentially expressed genes between female *Prmt7*$^{f/f}$ and *Prrx1-Cre; Prmt7*$^{f/f}$ groups,

indicating impacted biological processes. The red boxes indicate enriched pathways or biological processes related to osteogenesis and odontogenesis.

C. GO analysis illustrating the gene ontology categories, such as biological processes, cellular components, and molecular functions, enriched in the differentially expressed genes between female *Prmt7*$^{f/f}$ and *Prrx1-Cre; Prmt7*$^{f/f}$ groups. The red boxes indicate enriched pathways related to osteogenesis and odontogenesis.

D. Volcano plot showing the comparison of differentially expressed genes between female *Prmt7*$^{f/f}$ and *Sp7-Cre; Prmt7*$^{f/f}$ groups.

E. KEGG pathway analysis identifying enriched pathways in the differentially expressed genes between female *Prmt7*$^{f/f}$ and *Sp7-Cre; Prmt7*$^{f/f}$ groups. The red boxes indicate enriched pathways or biological processes related to osteogenesis and odontogenesis.

F. GO analysis illustrating the gene ontology categories enriched in the differentially expressed genes between female *Prmt7*$^{f/f}$ and *Sp7-Cre; Prmt7*$^{f/f}$ groups. The red boxes indicate enriched pathways related to osteogenesis and odontogenesis.

G. qRT-PCR results of *Prmt7* and *Pten* in mBMSCs from the femurs of six-week-old male and female mice.

H. Western blot images (left) of PRMT7 and PTEN in mBMSCs from the femurs of six-week-old male and female mice. Quantification (right) of relative PRMT7 and PTEN levels normalized to GAPDH.

I. qRT-PCR results of *PTEN* in PRMT7sh and control hBMSCs.

J. qRT-PCR results of *PRMT7* and *PTEN* in PRMT7sh and control DPSCs.

K. Representative Western blot images (left) of PTEN in PRMT7-overexpression and control hBMSCs. Quantification (right) of relative PTEN levels normalized to GAPDH.

L. qRT-PCR results of *PRMT7* and *PTEN* in PRMT7-overexpression and control hBMSCs.

M. Representative Western blot images (left) of PTEN in PRMT7-overexpression and control DPSCs. Quantification (right) of relative PTEN levels normalized to GAPDH.

N. qRT-PCR results of *PRMT7* and *PTEN* in PRMT7-overexpression and control DPSCs.

All data are mean ± SD, n = 3 biological replicates. (**, *P* <0.01, ***, *P* <0.001) (Independent samples t-test).

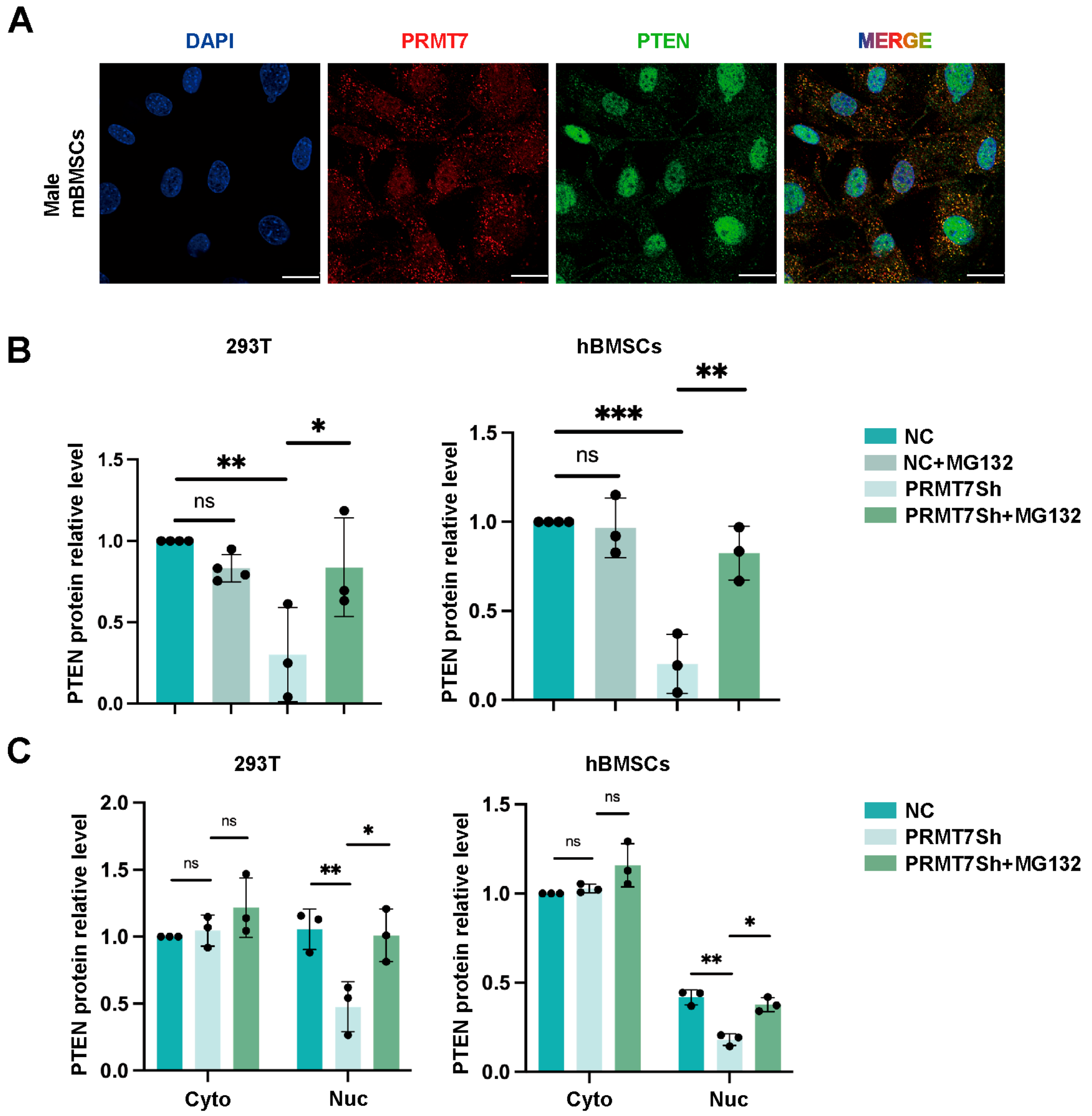

Figure S5: PRMT7 binds to and stabilizes PTEN in the nucleus, related Figure4.

A. Co-localization of PRMT7 and PTEN in male mBMSCs. Scale bar: 20 μm.

B. Quantification of relative PTEN levels in 293T and hBMSCs normalized to GAPDH, related to Figure4I.

C. Quantification of relative cytoplasmic and nuclear PTEN levels in 293T and hBMSCs normalized to a-tubulin and Lamin-B1 respectively, related to Figure4J.

n = 3 biological replicates. (ns, not significant, *, P<0.05, **, P <0.01, ***, P <0.001) (One-way ANOVA).

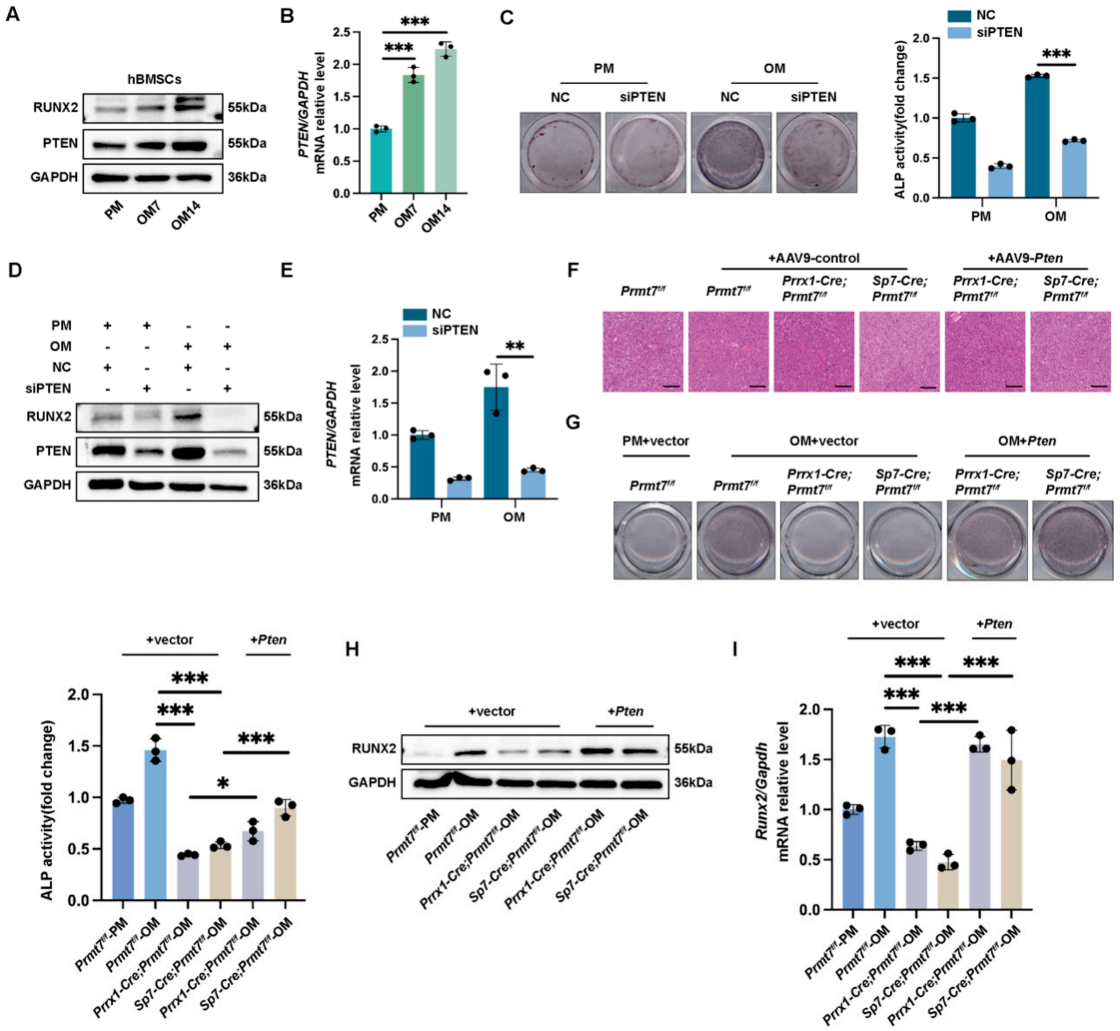

Figure S6: Bone loss in *Prmt7* CKO mice is mitigated by PTEN, related to Figure5.

A. Representative Western blot images of PTEN and RUNX2 in hBMSCs after 7 days and 14 days of osteogenic induction.

B. qRT-PCR results of *PTEN* in hBMSCs after 7 days and 14 days of osteogenic induction.

C. Staining and quantification of ALP in siPTEN and control hBMSCs after 7 days of osteogenic induction.

D. Representative Western blot images of RUNX2 and PTEN in siPTEN and control hBMSCs after 7 days of osteogenic induction.

E. qRT-PCR results of *PTEN* in siPTEN and control hBMSCs after 7 days of osteogenic induction.

F. H&E staining of liver sections from 12-week-old female *Prmt7*$^{f/f}$, *Prrx1-cre; Prmt7*$^{f/f}$ and *Sp7-cre; Prmt7*$^{f/f}$ mice after 6-week of AAV9-control or AAV9-*Pten* injection. Scale bar: 100 μm.

G. Staining and quantification of ALP in 6-week-old female *Prmt7*$^{f/f}$, *Prrx1-cre; Prmt7*$^{f/f}$ and *Sp7-cre; Prmt7*$^{f/f}$ mBMSCs with control or *Pten* plasmids after 7 days of osteogenic induction.

H. Representative Western blot images of RUNX2 in 6-week-old female *Prmt7*$^{f/f}$, *Prrx1-cre*; *Prmt7*$^{f/f}$ and *Sp7-cre; Prmt7*$^{f/f}$ mBMSCs with control or *Pten* plasmids after 7 days of osteogenic induction.

I. qRT-PCR results of *Runx2* in 6-week-old female *Prmt7*$^{f/f}$, *Prrx1-cre; Prmt7*$^{f/f}$ and *Sp7-cre; Prmt7*$^{f/f}$ mBMSCs with control or *Pten* plasmids after 7 days of osteogenic induction.

All data are mean ± SD, n = 3 biological replicates. (*, P<0.05, **, P<0.01, ***, P<0.001) (One-way ANOVA).

Table S1

Reagent/Resource	Source	Identifier or Catalog Number
Experimental Models		
C57BL/6J (M. musculus)	Cyagen Biological	C001089
C57BL/6JCya-*Prmt7*^{em1flox}/Cya (M. musculus)	Cyagen Biological	S-CKO-05852
Sp7-Cre (M. musculus)	Cyagen Biological	C001414
Prrx1-Cre (M. musculus)	Cyagen Biological	C001092
HEK293T	Scien-Cell	BFN60700191
hBMSCs	ScienCell Research Laboratories	7500

DPSCs	Creative Bioarray	CSC-C9774L
mBMSCs	Sample extraction	N/A
Recombinant DNA		
pcDNA3.1-CMV-mPTEN-3xflag- EF1-ZsGreen-T2A-Puro	Hanbio	2024030614
pcDNA3.1-CMV- EF1-ZsGreen-T2A-Puro	Hanbio	2024030615
HBAAV2/9-m-PTEN-3xflag-ZsGreen	Hanbio	89030815
HBAAV2/9-ZsGreen	Hanbio	89030814
pCI-neo-(HA)3-ubiquitin	Addgene	196991
pcDNA3.1(+) -PRMT7-Myc-Amp+	Tsingke Biotech Co., Ltd.	BJ0141952-1
pcDNA3.1(+) -PRMT7(E144A, D147A, E153A)-Myc-Amp+	Tsingke Biotech Co., Ltd.	BJ0147058-1
pcDNA3.1(+) -control-Amp+	Tsingke Biotech Co., Ltd.	202404161139
pcDNA3.1(+) -PTEN-3xflag-Amp+	Tsingke Biotech Co., Ltd.	202404161137
Antibodies		
Mouse monoclonal anti-GAPDH	Proteintech	60004-1-Ig
Rabbit monoclonal anti-Lamin B1	Cell Signaling Technology	D9V6H
Rabbit monoclonal anti-RUNX2	Cell Signaling Technology	12556
Rabbit polyclonal anti-Alpha Tubulin	Proteintech	11224-1-AP

Rabbit monoclonal anti-PRMT7	Cell Signaling Technology	14762
Rabbit polyclonal anti-PRMT7	Invitrogen	PA5-30748
Rabbit monoclonal anti-PTEN	Cell Signaling Technology	9188
Mouse monoclonal anti-PTEN	Invitrogen	32-5800
Mouse monoclonal anti-H3R2me1	Abcam	176844
Rabbit monoclonal anti-H3	Cell Signaling Technology	4499
Rabbit polyclonal anti-IgG	Cell Signaling Technology	2729
Rabbit monoclonal anti-HA	Cell Signaling Technology	3724
Oligonucleotides and other sequence-based reagents	Source	Sequence
PTEN siRNA	Hanbio	forward: CUAGAACUUAUCAAACCCUUUTT; reverse: AAAGGGUUUGAUAAGUUCUAGTT
NC siRNA	Hanbio	forward: UUCUCCGAACGUGUCACGU; reverse: ACGUGACACGUUCGGAGAATT
LV3-NC	GenePharma	TTCTCCGAACGTGT

		CACGT
LV3-PRMT7-Homo-1308	GenePharma	GGATGTGGTCGTG GAACAAGC
LV5-PRMT7 homo	GenePharma	190902CZ
LV5-NC	GenePharma	190627CZ
qPCR Primers	Forward	Reverse
GAPDH	CTGGGCTACACTGAGC ACC	AAGTGGTCGTTGA GGGCAATG
PRMT7	AGGCGGTTTGAACCTCT GAC	TGGTGGGCTACCA GATAGAGC
RUNX2	CCGCCTCAGTGATTTAG GGC	GGATCTGTAATCT GACTCTGTCC
PTEN	AGGGACGAACTGGTGT AATGA	CTGGTCCTTACTTC CCCATAGAA
Prmt7	GCCAGGTCATCCTATGC CG	GCCAATGTCAAGA ACCAAGGC
Runx2	CCGCACGACAACCGCA CCAT	CGCTCCGGCCCAC AATCTC
Fam229b	ATGCCTTTTCGGTTTGG GAC	CCGGTACAGGAAG CACTAGAG
Fsd1l	AAGGAAAGCATGATTA GCACCA	TGACTGAGCTGAC TCTGTAACT
Gapdh	GGTGAAGGTCGGTGTG AACG	CTCGCTCCTGGAA GATGGTG
Pten	TGGATTCGACTTAGACT TGACCT	GCGGTGTCATAAT GTCTCTCAG
ChIP qPCR Primers	Forward	Reverse
PTEN	GTTCACGTTCAGCACGC TCG	AACTCGGCTCGTTT GCCCTA

Pten	AGTTCCCCAACTAGGACCACA	GTCGGAACTACTTTCAGGAGGA
Genotyping	Forward	Reverse
Prmt7-flox	AGAAGAACATCTGCTCTAAGGTGC	ACAATTTTGTGGAAGTTGGTGCTCT
Prrx1-cre	GCTCTGATGTTGGCAAAGGGGT	AACATCTTCAGGTTCTGCGGG
Sp7-cre	TACCAGAAGCGACCACTTGAGC	GCACACAGACAGGAGCATCTTC
Chemicals, Enzymes and other reagents		
Protein A/G Magnetic Beads	MedChenExpress	HY-K0202
Anti-Flag Magnetic Beads	MedChenExpress	HY-K0207
Proteinase K (20 mg/ml)	Cell Signaling Technology	10012
MG132	APE×BIO	A2585
IP lysis buffer	Epizyme Biotech	PC105
Protease/Phosphatase inhibitors	NCM Biotech	P002
Triton™ X-100	MERCK	9036-19-5
TRIzol reagent	Invitrogen	15596018
SYBR Green Master Mix	YEASEN	11171ES03
Radio-immunoprecipitation assay (RIPA) lysis buffer	Solarbio	R0010
Phosphate buffer saline (PBS)	Beyotime	C0221A
Fetal bovine serum (FBS)	Gibco	A5256701
Penicillin/streptomycin	Gibco	15070063
Vitamin C	Sigma-Aldrich	V-047

β-glycerophosphoric acid	Sigma-Aldrich	154804-51-0
Dexamethasone	Sigma-Aldrich	265005
DAPI (4',6-Diamidino-2-Phenylindole, Dilactate)	BioLegend	422801
Lipofectamine 3000	Invitrogen	L3000008
Opti-MEM	Gibco	31985062
Polyvinylidene fluoride (PVDF) membranes	Millipore	3010040001
TBST	Servicebio	G0004-500ML
Collagenase, Type 2	Cell Signaling Technology	77336
Critical commercial assays		
NE-PER™	Thermo Scientific	78835
GenoCruz DNA Purification Kit	Santa Cruz Biotec	sc-45046
PrimeScript RT Reagent Kit	TaKaRa	RR047Q
BCA protein assay kit	Thermo Fisher Scientific	23227
BCIP/NBT Alkaline Phosphatase Color Development Kit	Beyotime	C3206
Alkaline phosphatase assay kit	Jiancheng Bioengineering	A059-2-2
Hematoxylin-Eosin (HE) Stain Kit	Solarbio	G1120
Modified Masson's Trichrome Stain Kit	Solarbio	G1346
Toluidine Blue O Solution	Solarbio	G3661
Endofree Maxi Plasmid Kit	TIANGEN	DP117

Software	
GraphPad software	https://www.graphpad.com
ImageJ	https://imagej.net/ImageJ
CT vox (3.3.0 r1383)	http://www.bruker microct.com
Data Viewer (1.7.0.1)	http://www.bruker.com
CaseViewer (2.4)	https://www.3dhistech.com/
Illustrator (24.3)	https://www.adobe.com/
Equipment	
SkyScan 1276 system	Bruker MicroCT, Kontich, Belgium
Confocal Microscope	Olympus Corporation, Tokyo, Japan
Western Blot Electrophoresis System	Bio-Rad Laboratories, Hercules, CA, USA
Real-time PCR system	Applied Biosystems, Foster City, CA, USA
Gel Imaging System	Clinx Science Instruments Co., Ltd, Shanghai, China
Gradient Thermal Cycler	Eppendorf AG, Hamburg, Germany
Microplate Reader	BioTek Instruments, Winooski, VT, USA
Covaris M220 Focused-ultrasonicator	Covaris Inc., Woburn, MA, USA
CO_2 Incubator	Esco Micro Pte. Ltd., Singapore
Biological Safety Cabinet	Thermo Fisher Scientific, Waltham, MA, USA
High-Speed Centrifuge	Eppendorf AG, Hamburg, Germany
NanoDrop 8000	Thermo Fisher Scientific, Wilmington, DE, USA
